# Circulating immune cell landscape and T‐cell abnormalities in patients with moyamoya disease

**DOI:** 10.1002/ctm2.1647

**Published:** 2024-04-02

**Authors:** Peicong Ge, Chuming Tao, Wenjing Wang, Qiheng He, Chenglong Liu, Zhiyao Zheng, Siqi Mou, Bojian Zhang, Xingju Liu, Qian Zhang, Rong Wang, Hao Li, Dong Zhang, Jizong Zhao

**Affiliations:** ^1^ Department of Neurosurgery Beijing Tiantan Hospital Capital Medical University Beijing China; ^2^ China National Clinical Research Center for Neurological Diseases Beijing China; ^3^ Center of Stroke Beijing Institute for Brain Disorders Beijing China; ^4^ Beijing Key Laboratory of Translational Medicine for Cerebrovascular Disease Beijing China; ^5^ Beijing Translational Engineering Center for 3D Printer in Clinical Neuroscience Beijing China; ^6^ Beijing Institute of Hepatology Beijing YouAn Hospital Capital Medical University Beijing China; ^7^ Department of Neurosurgery National Center of Gerontology Beijing Hospital Beijing China

**Keywords:** immune dysfunction, landscape, moyamoya disease, T‐cell abnormalities

## Abstract

**Background:**

Moyamoya disease (MMD) stands as a prominent cause of stroke among children and adolescents in East Asian populations. Although a growing body of evidence suggests that dysregulated inflammation and autoimmune responses might contribute to the development of MMD, a comprehensive and detailed understanding of the alterations in circulating immune cells associated with MMD remains elusive.

**Methods:**

In this study, we employed a combination of single‐cell RNA sequencing (scRNA‐seq), mass cytometry and RNA‐sequencing techniques to compare immune cell profiles in peripheral blood samples obtained from patients with MMD and age‐matched healthy controls.

**Results:**

Our investigation unveiled immune dysfunction in MMD patients, primarily characterized by perturbations in T‐cell (TC) subpopulations, including a reduction in effector TCs and an increase in regulatory TCs (Tregs). Additionally, we observed diminished natural killer cells and dendritic cells alongside heightened B cells and monocytes in MMD patients. Notably, within the MMD group, there was an augmented proportion of fragile Tregs, whereas the stable Treg fraction decreased. MMD was also linked to heightened immune activation, as evidenced by elevated expression levels of HLA‐DR and p‐STAT3.

**Conclusions:**

Our findings offer a comprehensive view of the circulating immune cell landscape in MMD patients. Immune dysregulation in patients with MMD was characterized by alterations in T‐cell populations, including a decrease in effector T‐cells and an increase in regulatory T‐cells (Tregs), suggest a potential role for disrupted circulating immunity in the aetiology of MMD.

## Graphical abstract

1. Single‐cell RNA sequencing, mass cytometry and RNA‐sequencing were used to compares immune profiles in MMD patients and healthy controls.

2. Immune dysregulation in patients with MMD was characterized by alterations in T‐cell populations, including a decrease in effector T‐cells and an increase in Tregs.

3. Disrupted circulating immunity might play a role in the aetiology of MMD.

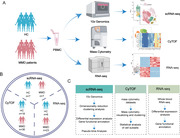

## 1 INTRODUCTION

Moyamoya disease (MMD) is a rare cerebrovascular disorder characterized by the narrowing or blockage of the internal carotid arteries and the development of abnormal collateral blood vessels at the base of the brain.^1^, ^2^ It predominantly affects children and adolescents in East Asian populations and is a leading cause of stroke in these populations.^3^

The exact cause of MMD is not yet fully understood, but multiple factors, including immune system dysregulation, inflammation, genetics and other factors, have been proposed as potential contributors to the development and progression of the disease.^4^, ^5^ One significant genetic factor associated with MMD is the RNF213 p.R4810K variant, which is highly prevalent in Japanese and Korean populations.^6^, ^7^, ^8^ However, it is worth noting that p.R4810K variants are found less frequently in China. This suggests that there may be other factors that contribute to the development of MMD in China. A growing body of research has shown that dysregulated inflammation and autoimmune responses contribute to the development of MMD.^9^, ^10^, ^11^, ^12^, ^13^ However, the lack of exhaustive research on MMD peripheral blood mononuclear cells (PBMCs) has hindered further understanding of the role of dysregulated inflammation in the aetiology of the disease.

In this study, we combined single‐cell RNA sequencing (scRNA‐seq) and mass cytometry (CyTOF) to analyse the properties of PBMCs in individuals with MMD. By examining the transcriptomic and proteomic profiles of immune cells, we aimed to gain insights into the immune dysfunction associated with MMD, particularly focusing on T‐cell (TC) clusters. These findings contribute to our understanding of MMD and may provide new insights for the development of new treatment strategies.

## 2 METHODS

### 2.1 Patients

All subjects included in the study were recruited from the Department of Neurosurgery, Beijing Tiantan Hospital. All patients were derived from the Early Screening and Identification for Panvascular Diseases Registry study (ClinicalTrials.gov Identifier: NCT05485870). MMD was diagnosed using digital subtraction angiography, following the Japanese guidelines published in 2012^14^: The diagnosis indicated stenosis or occlusion in the terminal internal carotid arteries, as well as the proximal middle and anterior cerebral arteries, with bilateral involvement.

### 2.2 Isolation of PBMCs for scRNA‐seq and CyTOF

Peripheral venous blood samples were collected in the morning from both healthy controls (HCs) and patients with MMD. Prior to the blood sampling, all subjects had fasted for a minimum of 12 h. TGFβ1 levels in plasma were measured using a commercially available ELISA kit from R&D Systems, located in. The blood samples were treated with the Ficoll‐Paque medium, which is commonly used for isolating PBMCs. The Ficoll‐Paque medium helps separate PBMCs from other components of blood based on their density. To maintain the viability and yield of the isolated PBMCs, the blood samples underwent heparinization and were processed through conventional density gradient centrifugation methods. Density gradient centrifugation is performed by overlaying the blood sample onto a medium with varying densities and subjecting it to centrifugal force. This results in the separation of different cell types based on their density.

After the isolation of PBMCs, the viability and quantity of the cells were assessed using Trypan Blue staining. In this study, it was found that the cell viability of the PBMCs in single‐cell suspensions exceeded 90%. For subsequent analyses, such as scRNA‐seq and CyTOF, a fraction of the isolated PBMCs from each sample was used. These techniques enable the characterization of the transcriptomic and proteomic profiles of individual cells, respectively. By following these standardized procedures, the study ensured the isolation of viable PBMCs and allocated appropriate samples for the subsequent analyses, allowing for comprehensive profiling of immune cells in both healthy individuals and patients with MMD.

### 2.3 Single‐cell preparation and sequencing

In the study, each sample necessitated around 12000 cells. The researchers utilized the Chromium Next GEM Single Cell 3′ GEM, Library & Gel Bead Kit v3.1 from 10× Genomics for single‐cell capturing and library construction, following the manufacturer's guidelines. The procedure was initiated by loading the cell suspension, barcoded gel beads and partitioning oil onto the 10× Genomics Chromium Chip, resulting in the creation of single‐cell gel beads in emulsion (GEMs). Inside each GEM, the captured cells were lysed, and their transcripts were barcoded through reverse transcription. The cDNA, along with cell barcodes, was subsequently subjected to PCR amplification. It is worth noting that no cell hashing, a technique for multiplexing samples, was applied in this study. Following library construction, the prepared libraries underwent sequencing on an Illumina NovaSeq platform, resulting in the generation of paired‐end reads of 2 × 150 bp^2^. The study included seven patients diagnosed with MMD, recruited from the centre.

### 2.4 Single‐cell RNA‐seq data‐processing flow

For the original sequences obtained from sequencing, we annotated them with the human reference version GRCh38. For HCs, their raw gene expression matrices were sourced from the Gene Expression Omnibus (GEO) database under the accession number GSE165080. Careful selection of controls was ensured, considering both age and gender for appropriate matching. To compare the differences in PBMC cell states between MMD and HCs, we applied integration methods outlined in Stuart et al. (https://satijalab.org/seurat/v3.0/integration.html).^15^ In brief, 2000 features with substantial cell‐to‐cell variation were identified. Subsequently, the ‘anchors’ among individual datasets were determined using the FindIntegrationAnchors function. Subsequently, the identified anchors were utilized as input for the IntegrateData function, facilitating the creation of a batch‐corrected expression matrix that included all cells. This approach enabled the integration and joint analysis of cells originating from various datasets. Genes expressed in fewer than 3 cells and cells with fewer than 100 or more than 5000 genes, as well as those with a mitochondrial gene percentage surpassing 25%, were excluded to eliminate partial cells and doublets.^16^, ^17^, ^18^ Following the exclusion of low‐quality cells, we use the R package ‘Seurat’ to perform standardization, dimensionality reduction and clustering analysis on single‐cell data. For the specific process, we refer to Seurat‐Guided Clustering Tutorial (https://satijalab.org/seurat/v3.0/pbmc3k_tutorial.html).

### 2.5 Differential gene expression analysis and functional enrichment

To identify genes with differential expression, we employed the FindMarkers function in Seurat, specifying the default parameter ‘test.use = wilcox’. The Benjamini–Hochberg method estimated the false discovery rate (FDR). The criteria for selecting differentially expressed genes (DEGs) encompassed a minimum log2(fold change) of 0.5 and a maximum FDR value of 0.01. Following this, we performed functional enrichment analysis on the identified DEGs using the Metascape webtool (www.metascape.org).

### 2.6 Defining cell state scores

To assess the expression of gene sets within a single cell, we used a cell score, which we used to refer to the study by Zhang et al.^19^ We employed distinct markers to delineate various cellular states, using CCR7, TCF7, LEF1 and SELL for naive cells, and PRF1, IFNG, GNLY, NKG7, GZMB, GZMA, GZMH, KLRK1, KLRB1, KLRD1, CTSW and CST7 for cytotoxicity. Exhaustion was characterized by LAG3, TIGIT, PDCD1, CTLA4, HAVCR2 and TOX, whereas endothelial‐mesenchymal transition (EndMT) was assessed using PDGFB, TGFB1, EGF, HGF, IGF1 and CXCL12.

### 2.7 Trajectory analysis

We used Monocle2 to analyse lineage differentiation of cell subtypes with potential developmental relationships. Initially, the top 2000 highly variable genes within CD4^+^T, CD8^+^T and regulatory TC (Treg) subsets were selected using the Seurat 3.0.0 FindVariableFeatures function, serving as the basis for ordering cells. Subsequently, we utilized the ‘DDRTree’ R package to construct tree‐like trajectories, providing insights into cellular development. For visual representation along the developmental trajectory, a heatmap was generated, specifically showcasing marker genes of CD4^+^T, CD8^+^T and Treg subtypes. Utilizing CD4^+^T, CD8^+^T and Treg subtypes, we inferred the evolutionary paths, utilizing the top 1000 highly variable genes to define the trajectories. Additionally, we employed Slingshot to unravel the developmental trajectory of CD4^+^T, CD8^+^T and Treg cells.

### 2.8 CyTOF live cell barcoding and surface staining

To minimize intersample staining variability, handling time and antibody consumption, we employed a live cell barcoding approach. Cell surface staining was performed using the Maxpar Direct Immune Profiling Assay by Fluidigm, incorporating monoclonal anti‐human antibodies (detailed in Table S1). Following barcoding, samples were washed and stained with Cisplatin‐195pt. The fixed samples were then treated with 1.6% paraformaldehyde (PFA) in PBS and resuspended in MaxPar Cell Staining buffer. After washing, the fixed sample was placed on a shaker with a mixture of surface antibodies and incubated at 37°C for 30 min. To preserve staining, the samples were stored overnight at 4°C in a solution containing 2% formaldehyde in PBS and an intercalator (iridium 191/193).

Use the Cytobank to manual gating each cell subtype from each sample. Perform sinusine normalization of the data exported from Cytobank to ensure an unbiased determination of clustering. For high‐dimensional data analysis, The R package ‘cytofkit’ (v1.10.0) is used for the progressive PhenoGraph flow, and the parameter selects the default value. In summary, the data underwent thorough preprocessing and analysis, including gating, normalization, clustering and visualization (default values for all parameter selections), to identify and characterize distinct cell populations. The subsequent statistical analysis using the Wilcoxon rank‐sum test helped to identify significant differences among groups, providing valuable insights into the cell‐level changes associated with MMD.

### 2.9 RNA extraction and library construction

For the RNA‐seq analysis, PBMCs were collected from 23 patients and 6 HCs. RNA is then extracted to construct a sequencing library. Subsequently, the prepared libraries underwent paired‐end sequencing on the Illumina NovaSeq 6000 platform. In addition, subsequent sequencing of the libraries was carried out on the Illumina HiSeq platform, yielding paired‐end reads with a length of 150 base pairs. By employing this high‐throughput sequencing approach, the researchers were able to obtain comprehensive RNA expression profiles from the PBMCs of both the patients and HCs. These data would allow them to conduct in‐depth analysis and explore the transcriptomic differences between the two groups, potentially uncovering valuable insights related to the studied conditions.

### 2.10 Statistical analysis

Statistical analysis was conducted using R/4.2.2. Data distribution was evaluated, and depending on normality, comparisons utilized either the Student *t*‐test (for two groups, parametric) or the Wilcoxon signed‐rank test (for two groups, paired) with two‐tailed *p* values, unless otherwise specified. Significance thresholds were established at *p* < .05 (*), *p* < .01 (**), *p* < .001 (***) and *p* < .0001 (***–) to indicate various levels of statistical significance.

## 3 RESULTS

### 3.1 Cohort characteristics and single‐cell analysis of PBMCs

To construct a comprehensive immune cell atlas delineating the immunological characteristics of individuals with MMD, we amalgamated scRNA‐seq, CyTOF and RNA‐seq data from PBMC suspensions across three distinct cohorts (Graphical Abstract). In scRNA‐seq cohort, comprising seven MMD patients in our centre and seven age–sex‐matched HCs. In the CyTOF cohort, which included 36 MMD patients and 18 age–sex‐matched HCs, CyTOF analysis was conducted. Additionally, in the RNA‐seq cohort, consisting of 23 MMD patients and 6 age–sex‐matched HCs, RNA‐seq was performed. In the CyTOF cohort, we have a total of 34 markers set up in our CyTOF panel. The expression of these marker proteins is used to determine the alteration of cell distribution in MMD patients. Subsequently, in the RNA‐seq cohort, we conducted an analysis to compare cellular distinctions between MMD patients and HCs. Furthermore, we conducted a comparison of plasma TGFβ1 levels in a cohort of 120 MMD patients and 35 age–sex‐matched HCs (Graphical Abstract). Comprehensive clinical information for both MMD patients and HCs is provided in Tables S1–S4.

### 3.2 Single‐cell transcriptional profiling of peripheral immune cells

**FIGURE 1:**
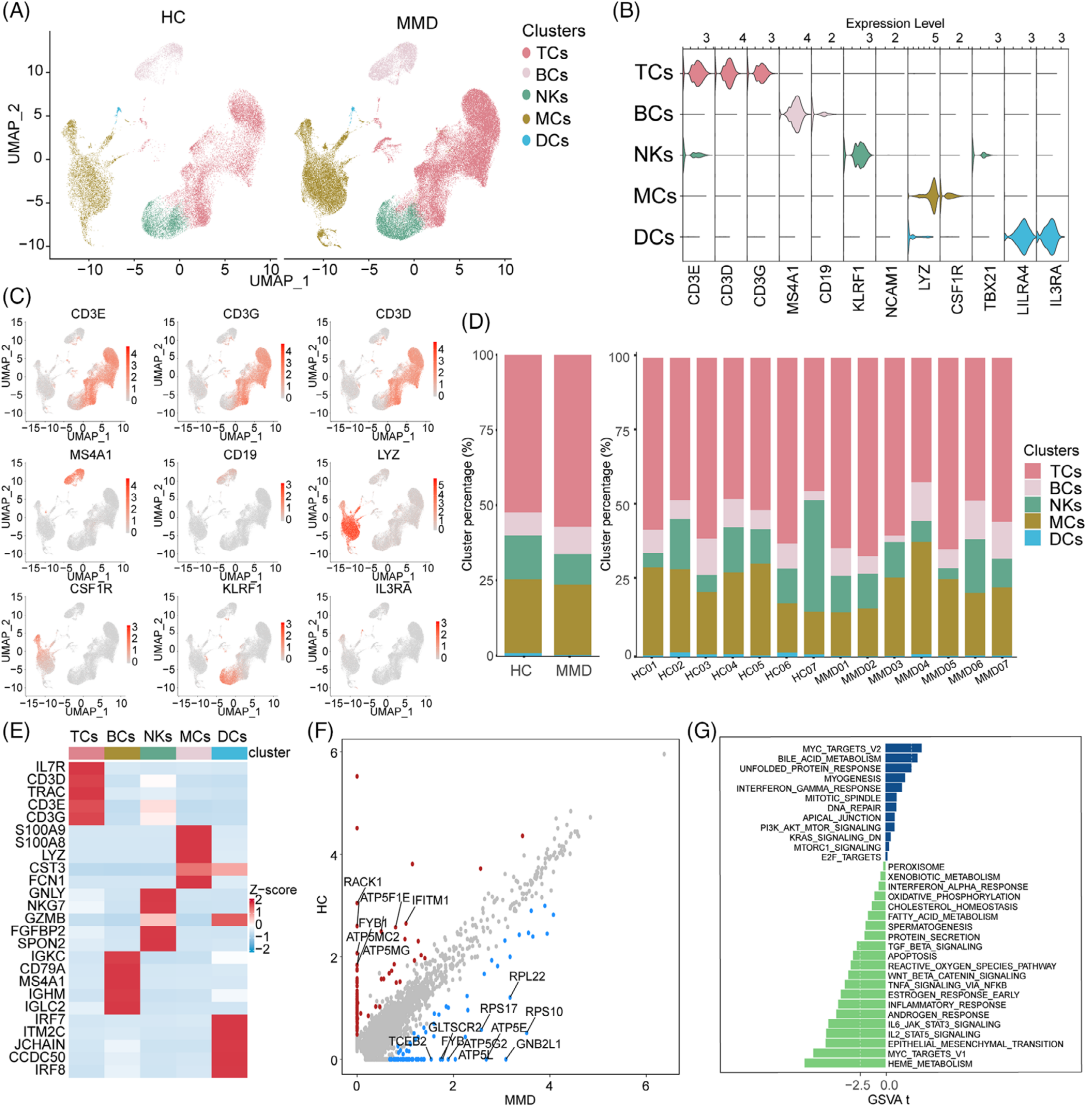
Single‐cell transcriptomic profiling of peripheral blood mononuclear cells (PBMCs) from single‐cell RNA sequencing (scRNA‐seq) cohort. (A) The uniform manifold approximation and projection (UMAP) projection of PBMCs derived from scRNA‐seq cohort. (B) Violin plots showing the expression distribution of selected canonical cell markers in the five cell subsets. (C) Cell markers are used to identify and verify the distribution of each cell subset. (D) The proportion of each cell subset as well as its distribution. Moyamoya diseases (MMDs) (*n*  =  7) and healthy controls (HCs) (*n*  =  7). (E) Heatmap shows the top five highly expressed genes in each cell subset. *z*‐score: −2 (light blue) to 2 (red). (F) The scatter plot shows the distribution of differentially expressed genes (DEGs) between the MMD and HC groups. (G) gene set variation analysis (GSVA) analysis showed a significant enrichment of signalling pathways between the MMD and HC groups. *t* Values corrected for patient of origin.

In the scRNA‐seq cohort, we employed uniform manifold approximation and projection (UMAP) to capture the transcriptomes of five major cell types based on the expression of canonical gene markers (Figure 1A,B; Figure S1A,B). We identified the cell subsets of PBMCS. These cell types included TCs (CD3D, CD3E, CD3G), B cells (BCs) (CD19, MS4A1), natural killer (NK) cells (KLRF1), monocytes (MCs) (LYZ, CSF1R) and dendritic cells (DCs) (LILRA4, IL3RA) (Figure 1B). Cell markers are used to identify and verify the distribution of each cell subset (Figure 1C). According to the research results of scRNA‐seq, we also distinguished the aforementioned 5 types of cells in the CyTOF cohort (Figure S2A–C). Cell markers are used to identify and verify the distribution of each cell subset in the CyTOF cohort (Figure S2D). Notably, the distribution of cell subsets within these five lineages exhibited variations between the MMD and HC groups (Figure 1D; Figure S1C). The CyTOF data revealed elevated percentages of BCs (*p* = .0085, two‐sided Wilcoxon‐test) and MCs (*p* = .00064, two‐sided Wilcoxon‐test) in MMD patients, while indicating reduced percentages of NKs (*p* = .0015, two‐sided Wilcoxon‐test) and DCs (*p* = 0.01, two‐sided Wilcoxon‐test), in comparison to HCs (Figure S2E,F). Overall, patients with MMD showed significant changes in their peripheral immune cells.

### 3.3 Changes in five cell lineages function in patients with MMD

For a deeper exploration of the transcriptomic alterations in immune cells among MMD patients, we contrasted the expression profiles of MMD patients with those of HCs across diverse immune cell types (Figure 1E,F). We observed significant DEGs associated with PI3K/AKT/mTOR signalling, KRAS signalling, E2F targets and hypoxia in patients with MMD (Figure 1G). These findings provide insights into the transcriptomic alterations within the immune cell population in MMD patients.

To further explore the transcriptome and protein changes in innate immune cells of MMD patients, we conducted a comparison of expression patterns between MMD and HCs in five cell lineages (Figure S3A). In TCs, it was found that inflammation‐related genes such as TRAC, CCR7 and IL7R were prominently expressed in scRNA‐seq cohort (Figure S3B). In the CyTOF cohort, the MMD group exhibited elevated expression levels of CCR7, CCR10, CXCR3, CX3CR1, CD45RO, CD11b, CD11c, CD25, CD27, CD62L, HLA‐DR, p‐STAT3 and ERK compared to the HCs group. Conversely, the levels of CCR6, CXCR4, PD‐1, MYD88, NFκB, AKT and TGFβ were diminished in the MMD group relative to the HC group (Figure S3C). In BCs, we compared the expression profiles of BCs between MMD patients and HCs in scRNA‐seq cohort. The DEGs that showed the most significant enrichment in patients with MMD were involved in the adaptive immune system, VEGFA–VEGFR2 signalling pathway, immune effector processes and interferon signalling. In the CyTOF cohort, MMD group displayed heightened expression levels of CCR10, CXCR3, CX3CR1, CD45RA, CD45RO, CD11b, CD11c, CD25, CD27, CD62L, HLA‐DR, p‐STAT3, ERK and TGFβ compared to the HC group. Conversely, the levels of CCR6, CXCR4, PD‐1, TLR4, MYD88, NFκB and AKT were diminished in the MMD group relative to the HC group (Figure S4A–D).

In NKs, we observed DEGs associated with immune system processes, biological regulation, metabolic processes and regulation of biological processes that were significantly enriched in MMD patient scRNA‐seq cohort. In the CyTOF cohort, the MMD group exhibited elevated expression levels of CCR7, CXCR3, CXCR4, CX3CR1, CD45RO, CD11b, CD11c, CD27, CD62L, HLA‐DR, ERK and TGFβ compared to the HC group. Conversely, the levels of CD127, PD‐1, MYD88, NFκB and AKT were reduced in the MMD group relative to the HC group (Figure S5A–D). In MCs, we identified significant DEGs that were involved in the IL‐17 signalling pathway, cell killing and regulation of apoptotic signalling pathway in patients with MMD in scRNA‐seq cohort. In the CyTOF cohort, the MMD group displayed increased expression levels of CD14, CXCR3, CX3CR1, CD45RA, CD45RO, CD11b, CD11c, CD25, CD27, CD62L, HLA‐DR and p‐STAT3 in comparison to the HC group. Conversely, the levels of CCR6, CXCR4, PD‐1, TLR4, MYD88, NFκB and AKT were reduced in the MMD group relative to the HC group (Figure S6A–D). In DCs, we identified significant DEGs that were involved in the immune effector process, cytokine signalling in immune system and cellular response to cytokine stimulus. In the CyTOF cohort, the MMD group exhibited elevated expression levels of CCR7, CD14, CXCR3, CXCR4, CX3CR1, CD45RA, CD45RO, CD11b, HLA‐DR, ERK, p‐STAT3, p‐STAT4 and TGFβ compared to the HC group. Conversely, the levels of CCR6, PD‐1, MYD88, NFκB and AKT were reduced in the MMD group relative to the HC group (Figure S7A–D). These findings indicate consistent characteristics of innate immune cells in response to MMD.

### 3.4 Compositional analysis reveals T‐cell abnormalities in MMD

**FIGURE 2:**
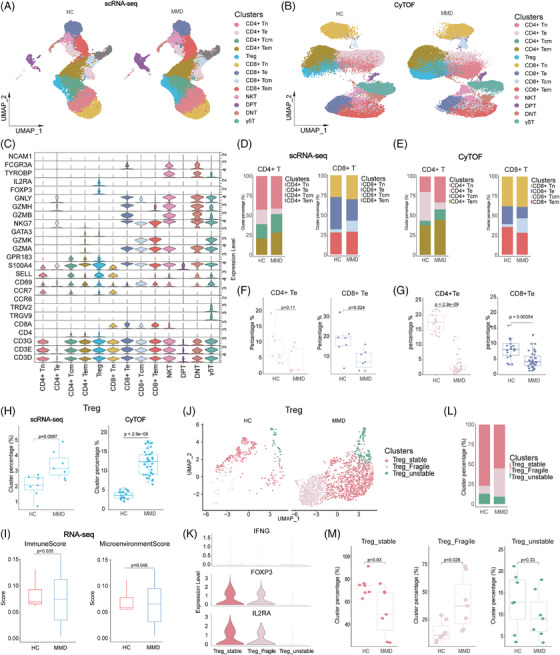
Compositional analysis reveals T‐cell abnormalities in Moyamoya disease (MMD). (A) The uniform manifold approximation and projection (UMAP) projection shows the distribution of 560099 T cells from scRNA‐seq cohort after dimensionality reduction clustering. (B) The UMAP projection shows the distribution of 56099 T cells from mass cytometry (CyTOF) cohort after dimensionality reduction clustering. (C) The violin plot illustrates the expression of common cellular markers of 13 T‐cell subsets. (D and E) Average proportion of CD4^+^T and CD8^+^T cell subsets in single‐cell RNA sequencing (scRNA‐seq) cohort (D) and CyTOF cohort (E). (F and G) The difference of CD4^+^Te and CD8^+^Te subsets in scRNA‐seq cohort (F) and CyTOF cohort (G). (H) The dot plot shows the imbalance of regulatory T cell (Treg) proportions between the MMD and healthy control (HC) groups in the scRNA‐seq cohort and the CyTOF cohort. (I) The difference of immuneScore and microenviromentScore between MMDs (*n* = 23) and HCs (*n* = 6). (J) UMAP projections of Treg cell derived from scRNA‐seq data. (K) Violin diagram showing the marker genes of Treg cells and the expression of inflammatory cytokines. (L) Distribution of Treg cell subtypes in scRNA‐seq cohort. (M) The dot plot shows the imbalance of Treg cell subtypes proportions between the MMD and HC groups in the scRNA‐seq cohort.

To categorize each cell subpopulation without bias, we independently performed reclustering of TCs from PBMCs in the two groups. Then, 13 TC subsets were distinguished by reference to the relative expression of TC markers (Figure 2A). These subtypes encompassed four variations of CD4^+^T cells (CD3E^+^CD4^+^), Treg cells (IL2RA^+^FOXP3^+^), four variations of CD8^+^T cells (CD3E^+^CD8A^+^), NKT cells (CD3E^+^NCAM1^+^), CD4^+^CD8^+^double‐positive TCs (DPT, CD3E^+^CD4^+^CD8A^+^), CD4^−^CD8^−^double‐negative TCs (DNT, CD3E^+^CD4^+^CD8A^+^) and γδT cells (TRDC^+^).

These clusters consisted of four types of CD4^+^T cells, including naive CD4^+^T cells (CD4^+^Tn, CCR7^+^SELL^+^), effector memory CD4^+^T cells (CD4^+^Tem, GZMA^+^S100A4^+^GPR183^+^), central memory CD4^+^T cells (CD4^+^Tcm, S100A4^+^GPR183^+^CCR7^+^SELL^+^) and effector CD4^+^T cells (CD4^+^Te, GZMA^+^GNLY^+^NKG7^+^). Similarly, the CD8^+^T cell clusters included naive CD8^+^T cells (CD8^+^Tn, CCR7^+^SELL^+^), effector memory CD8^+^T cells (CD8^+^Tem, GZMA^+^S100A4^+^GPR183^+^), central memory CD8^+^T cells (CD8^+^Tcm, S100A4^+^GPR183^+^CCR7^+^SELL^+^) and effector CD8^+^T cells (CD8^+^Te, GZMA^+^GNLY^+^NKG7^+^) (Figure 2C). The distribution of each cluster across MMD and HCs offered insights into the characteristics of TC subsets (Figure S8B). To validate the changes in TC ratios, we performed single‐cell protein‐level analysis. Aligned with the cell subsets recognized in the scRNA‐seq outcomes, TCs were reclustered into 13 subpopulations in the CyTOF cohort: CD4^+^Tn, CD4^+^Te, CD4^+^Tcm, CD4^+^Tem, Treg, CD8^+^Tn, CD8^+^Te, CD8^+^Tcm, CD8^+^Tem, DPT, DNT, NKT and γδT cells (Figure 2B).

To examine how the cell‐type composition changed between MMD patients and HCs, we evaluated the proportions of each TC subsets. We observed that the percentages of CD4^+^Tn, CD4^+^Tcm, CD4^+^Tem, Treg and CD8^+^Tcm were elevated in MMD patients compared to HCs. On the other hand, CD4^+^Te, CD8^+^Tn, CD8^+^Te, CD8^+^Tem and γδT cells showed significant decreases in MMD patients, whereas DNT, DPT, CD4^+^Tem and NKT did not exhibit significant changes between the two groups (Figure S8A,B; Figure S9A–E). Cell markers are used to identify and verify the distribution of each TC subset in the CyTOF cohort (Figure S9D). An augmentation in the count of Treg cells with immunosuppressive function (Figure 2H), accompanied by a reduction in the population of cytotoxic TCs responsible for killing (Figure 2F,G). Our findings strongly suggest a suppression state of peripheral immunity. Additionally, the CD8+Te cells displayed high expression of TBX21 (Figure S8C). On the contrary, CD4+Tem cells displayed features reminiscent of type 2 helper T (Th2) cells, characterized by elevated expression of GATA3 (Figure S8C). These findings highlight the alterations in TC populations as a key aspect to investigate changes in the immune microenvironment of MMD.

In MMD, the most notable alteration is the significant increase in Treg cells. Pathological angiogenesis is a prominent feature of MMD, and Treg cells play a crucial role in regulating this process.^20^ The classification of Treg has been studied in detail.^21^, ^22^, ^23^, ^24^ Therefore, based on previous studies, we defined Treg cell subpopulations into three subpopulations: stable Tregs, unstable Tregs and fragile Tregs. The criteria for its definition are as follows: Stable Tregs express Foxp3 and possess suppressive function, whereas fragile Tregs express Foxp3 but exhibit decreased expression of inhibitory factors like IL2RA, resulting in limited or no suppressive function and secretion of IFN‐γ; unstable Tregs do not express Foxp3 and lack suppressive function (Figure 2K). In addition, TGFβ1, TNFA and ICAM1 were expressed significantly in fragile and stable types (Figure 2J,K; Figure S8 D,E). Notably, in patients with MMD, there was an increase in the proportions of fragile Tregs compared to HCs, whereas the proportion of stable Tregs decreased (Figure 2M); this also explains the low proportion of effector TCs and the immunosuppression of MMD patients. At the same time, it was also corroborated by the less expression of chemokines CCR6, CXCR4, NF  κB and AKT in the CyTOF cohort in TCs. This suggests that the dysregulation of Treg cells may play a significant role in the development and progression of MMD.

### 3.5 Changes in T‐cell function in patients with MMD

**FIGURE 3:**
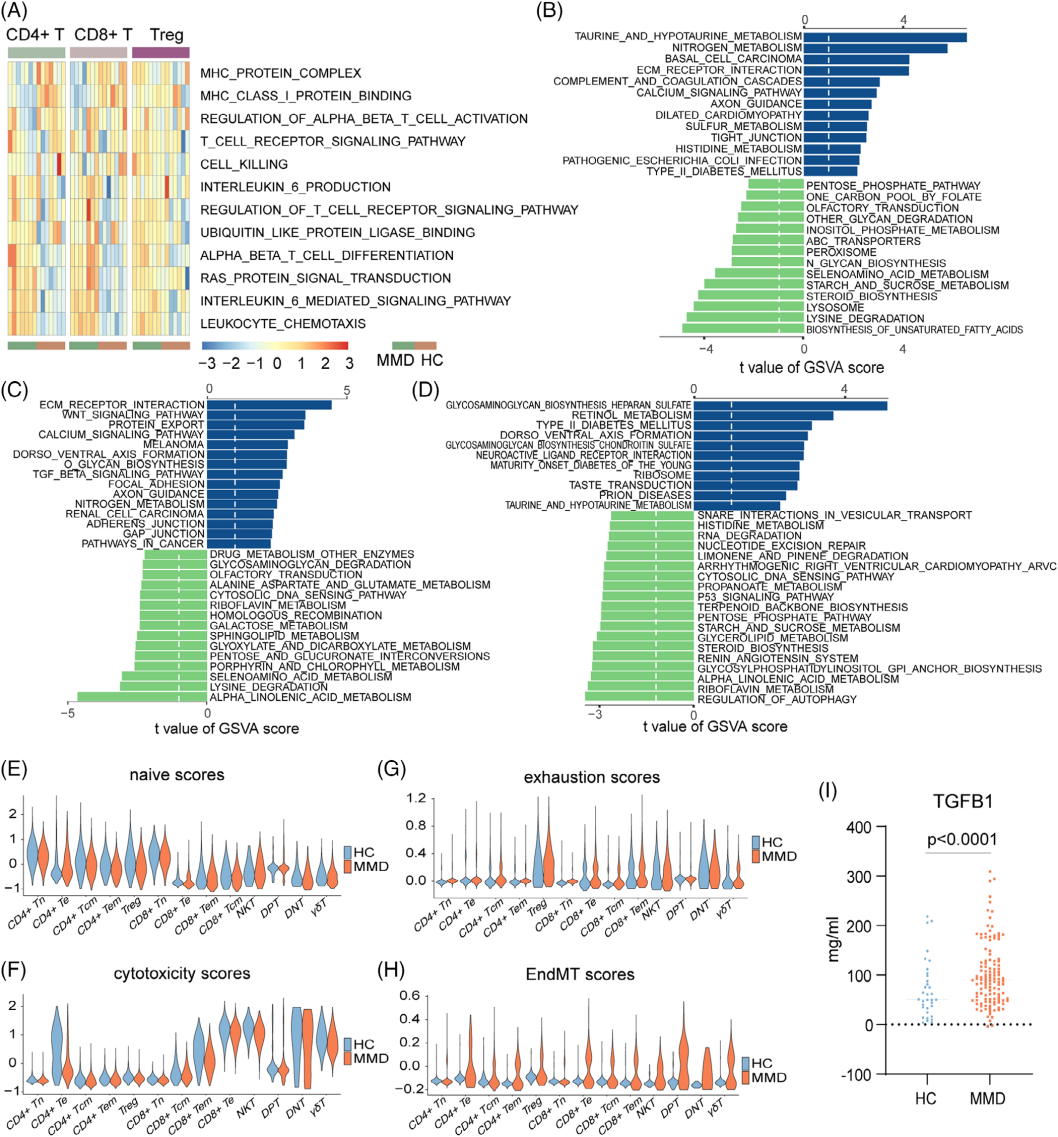
Changes in T‐cell function in patients with moyamoya disease (MMD). (A) GSVA score heatmaps of CD4^+^T, CD8^+^T and regulatory T cells (Tregs). (B) In CD4^+^T cell subset, GSVA enrichment analysis was performed between MMD and healthy control (HC) groups. (C) In CD8^+^T cell subset, GSVA enrichment analysis was performed between MMD and HC groups. (D) In Treg cell subset, GSVA enrichment analysis was performed between MMD and HC groups. (E–H) Violin plots of the naïve (E), cytotoxicity (F), exhaustion (G) and EndMT (H) scores across different TCs subsets. (I) ELISA detects the concentration of TGFB1 in serum in HCs and MMDs.

For a detailed exploration of the intrinsic functional changes in each TC subset, we performed a comprehensive analysis of CD4^+^T, CD8^+^T and Treg cells in both MMD patients and HCs in the scRNA‐seq cohort. We identified significant DEGs for each subset and utilized GSVA enrichment analysis to elucidate their specific alterations in biological functions and signalling pathways (Figure 3A–D; Figure S10A–F).The analysis results showed that deferentially expressed TC subgroups were significantly enriched in pathways related to metabolism, MHC signalling pathway, cell killing, leucocyte migration and endothelial–mesenchymal transition (EndMT) (Figure 3A,B; Figure S10G,H).

Leucocyte chemotaxis was significantly enriched in the MMD group, and metabolism‐related genes (GGT1, BAAT and ADO) and EndMT (TGFβ1, SMAD2, SMAD3 and SMAD4) tended to be enriched in CD4^+^T and CD8^+^T cell subgroups (Figure 3A–C; Figure S11A). Interestingly, similar results were obtained in the GSVA enrichment analysis for the RNA‐seq cohort, indicating significant alterations in the PBMCs of MMD patients compared to HCs, particularly in metabolism, JAK‐STAT signalling pathway and TGFβ signalling pathway (Figure S12B,C). Additionally, using an EndMT scoring system, we observed an overall higher trend in EndMT scores among MMD patients compared to HCs (Figure 3H). Furthermore, we enrolled 120 MMD patients and 35 age–sex‐matched HCs, and the serum TGFβ1 was significantly increased in MMD patients compared to HCs (Figure 3I), which was further validation of our single‐cell results. Furthermore, we screened DEGs between the MMD and HC groups in the three TC subtypes. In addition, we found that these genes were mainly enriched in immunomodulatory and angiogenic pathways (Figure S10H). In addition, we selected the genes related to PID CXCR4 pathway, VEGFA–VEGFR2 signalling, cytokine signalling in immune system and regulation of lymphocyte activation pathways and showed their expression through heatmaps, and most of the genes of these pathways are significantly upregulated in MMD patients (Figure S10I). These results may indicate that the dysregulation of immune cells in patients with MMD leads to changes in the immune microenvironment, which leads to pathological changes in the vascular changes of MMD.

Previous studies have confirmed a close correlation between human leucocyte antigen (HLA) and MMD.^25^, ^26^ Hence, we extensively examined the expression of HLA family genes within TC subsets. The figure displays the expression of HLA‐DRB5, HLA‐DPB1, HLA‐DMA, HLA‐DMB, HLA‐DPA1 and HLA‐DRB1 in CD4^+^T, CD8^+^T and Treg cells, and results show that HLA‐DMA and HLA‐DPA1 are particularly expressed in Treg cells (Figure S11A). Particularly, we observed higher expression of HLA‐A, HLA‐DMB, HLA‐DPB1 and HLA‐B in the RNA‐seq cohort of the MMD group (Figure S12A). The results suggest that the HLA family does have abnormal expression in the MMD population, and even though there are studies on the association between the HLA family and MMD, more research is still needed to explain the specific mechanism. In addition, genes associated with cell killing (TUBB, PTPN6, LYZ, ITGAM, IL7R, GZMB) exhibited higher expression levels in the CD4^+^T and CD8^+^T subgroups (Figure S11A). In CyTOF cohort, the expression levels of CCR10, CXCR3, CX3CR1, CD45RO, CD11b, CD11c, CD27, CD62L, HLA‐DR, p‐STAT3, and ERK were higher in MMD patients; the levels of CCR6, CXCR4, PD‐1, MYD88, NFκB, and AKT were lower than in the HC group in both CD4^+^T and CD8^+^T cells (Figure S11B,C). Combined with the previous analysis of the proportion of CD4^+^T and CD8^+^T, it is shown that the status of CD4^+^T and CD8^+^T cells in MMD patients is basically the same. In addition, the two cell subsets may play a synergistic role in the pathogenesis of MMD.

**FIGURE 4:**
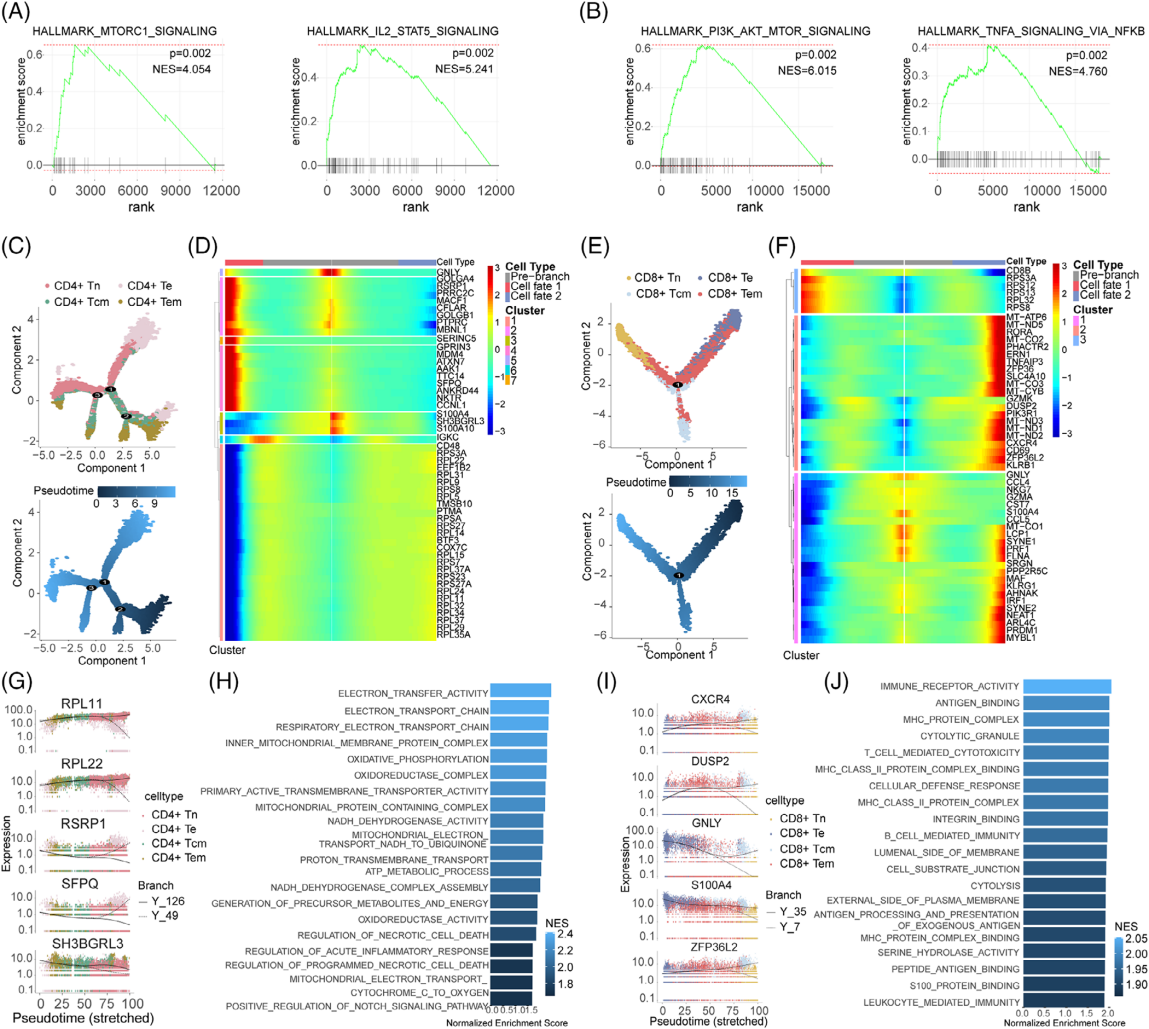
The trajectory analysis of the CD4^+^T and CD8^+^T cells in moyamoya disease (MMD). (A and B) gene set enrichment analysis (GSEA) analysis showed the pathways that the upregulated genes in CD4^+^T cell (A) and CD8^+^T cell (B) were enriched in. (C and E) Trajectory of CD4^+^T cell (C) and CD8^+^T cell (E). (D and F) Using pseudo‐time point 1 as the differentiation distinguishing point, the heatmaps show the upregulated gene heatmap between two cell subsets with different fates. (G) Top five genes differentially expressed in different clusters from CD4^+^T cell subset. (H) gene ontology (GO) analysis of upregulated genes in CD4^+^Te cell cluster in pseudo‐time analysis. (I) Top five genes differentially expressed in different clusters from CD8^+^T cell subset. (J) GO analysis of upregulated genes in CD8^+^Te cell cluster in pseudo‐time analysis.

The naive state of TCs reflects their potential for differentiation.^27^ TC exhaustion is characterized by a dysfunctional state where TCs lose robust effector functions and express multiple inhibitory receptors.^28^, ^29^ Therefore, we assessed the naive and exhaustion scores of various TC in both HCs and MMD patients. Among the subsets, CD4^+^Tn and CD8^+^Tn exhibited relatively higher naive scores. However, the CD4^+^Tn, CD8^+^Te, CD4^+^Tcm, CD4^+^Tem, CD8^+^Tn and Treg subsets in MMD patients displayed lower naive scores compared to their counterparts in HCs (Figure 4E). CD8^+^Te, CD4^+^Te and NKT demonstrated higher cytotoxicity scores than the other subsets, and HCs had the lowest cytotoxicity scores (Figure 4F). Furthermore, CD8^+^Te, CD4^+^Tcm, CD4^+^Tem and Treg subsets exhibited higher exhaustion scores than the other subsets, with HCs displaying the lowest exhaustion scores within these highly exhausted subsets (Figure 4G). These findings are supported by the reduced proportion of CD4^+^Te cells observed in MMD patients. The scores of these TC subpopulations not only further analyse and verify the status of their cells but also provide a reference for future research on how TCs affect the peripheral immune microenvironment of patients with MMD.

### 3.6 The trajectory analysis of the T cells in MMD

**FIGURE 5:**
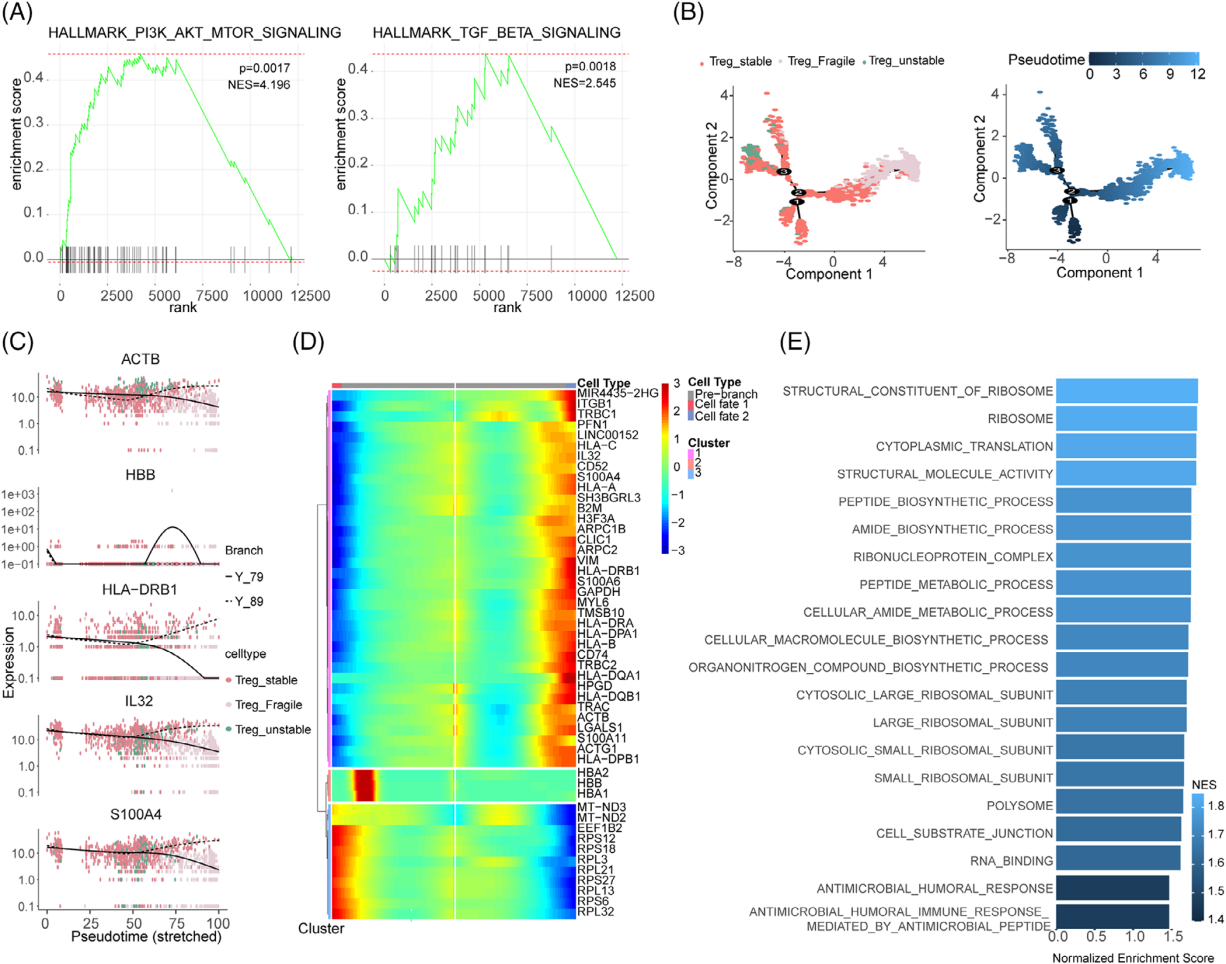
The trajectory analysis of the regulatory T cells (Tregs) in moyamoya disease (MMD). (A) GSEA analysis showed the pathways that the upregulated genes in Treg were enriched in. (B) Trajectory of Treg cells. (C) Top five genes differentially expressed in different clusters from Treg cell subset. (D) Using pseudo‐time point 1 as the differentiation distinguishing point, the heatmaps show the upregulated gene heatmap between two cell subsets with different fates. (E) GO analysis of upregulated genes in Treg cell cluster in pseudo‐time analysis.

To gain a deeper understanding of how these CD4^+^T cells, CD8^+^T cells and Treg cells subset may contribute to MMD, GSEA was performed. We found that in the CD4^+^T cell subset, genes highly expressed in patients with MMD are significantly enriched in MTORC1_SIGNALING and IL2_STAT5_signaling pathways, and in CD8^+^T cell subset, PI3K_AKT_MTOR_signaling and TNFA_signaling_via_NFκB pathways were significantly enriched (Figure 4A,B). In order to more clearly show the expression of these pathway genes, we used heatmaps to show their expression in different TC subsets and different individuals (Figure S13A–D). To gain further insights into the developmental progression of CD4^+^T and CD8^+^T cell subsets in MMD, trajectory analysis was conducted for both subsets (Figure 4C,E). Despite conducting a detailed analysis of the proportion and functional status of TCs, we observed a distinct bifurcated architecture in the cell trajectory, suggesting a divergence in transcriptional states (Figure 4C,E). Moreover, we identified five representative genes that play a role in determining the fate of CD4^+^T cells (RPL11, RPL22, RSRP1, SFPQ, SH3BGRL3) and CD8^+^T cells (CXCR4, DUSP2, GNLY, S100A4, ZFP36L2) (Figure 4G,I). CD4^+^Te cells (identified as clusters 2) at the root of the trajectory were mainly enriched in the regulation of acute inflammatory response and oxidoreductase activity. Although CD8^+^Te cells (corresponding to cluster 1) at the initial stage of the trajectory were predominantly enriched in immune receptor activity, MHC protein complex, S100 protein binding and leucocyte‐mediated immunity (Figure 4H,J). In addition, we conducted GSEA to Treg cell subsets; results showed that the MMD group was enriched in PI3K_AKT_MTOR_SIGNALING and TGF_BETA_SIGNALING pathways (Figure 5A), and the heatmaps show their expression in different TC subsets and different individuals in Treg (Figure S13E,F). The trajectory analysis of Tregs to further understand their developmental progression. The results revealed significant differences in the pseudo‐time distribution of the three Treg subgroups: stable Tregs, unstable Tregs and fragile Tregs, stable Tregs at beginning of the trajectory (Figure 5B), the five representative genes (ACTB, HBB, HLA‐DRB1, IL32, S100A4) are implicated in shaping the fate of Treg cells (Figure 5C,D). The fragile Tregs at the base of the trajectory were predominantly associated with RNA binding, cellular amide metabolic processes and cytoplasmic translation (Figure 5E). The above findings validate the TC differentiation trajectory, reinforcing the observed imbalance in the mentioned TC subsets. These results offer insights into the functional characteristics and signalling pathways associated with TCs in individuals with MMD.

## 4 DISCUSSION

MMD is now increasingly acknowledged as a condition primarily influenced by chronic inflammation, as indicated by previous research studies.^9^, ^11^, ^30^, ^31^, ^32^, ^33^ Circulating immune cells are believed to contribute to MMD and its progression. These immune cells are believed to play a role in MMD by infiltrating blood vessel walls, triggering the release of proinflammatory molecules. Furthermore, in addition, they may interact with vascular endothelial cells and promote aberrant alterations in vascular smooth muscle cells.^9^, ^12^, ^34^ We have observed immune dysfunction in MMD patients, specifically involving dysregulation of TC clusters, which was characterized by decreased effector TCs and increased regulatory TCs, along with decreased NKs, DCs and increased BCs, MCs. Notably, in patients with MMD, there was an increase in the proportions of unstable Tregs and fragile Tregs, whereas the proportion of stable Tregs decreased.

Dysregulation of TCs has been associated with the pathogenesis of numerous chronic inflammatory disorders.^35^, ^36^ Specifically, TCs have been shown to contribute to the development of atherosclerosis, a major risk factor for cerebrovascular disease.^37^, ^38^ Dysregulation of TCs can cause an immune system imbalance, leading to chronic inflammation and damage to the endothelial cells that line the blood vessels. The imbalance between pro‐ and anti‐inflammatory subsets of TCs, particularly, can contribute to the pathogenesis of cerebrovascular disease. Previous study found that the dysregulation of RNF213, a susceptibility gene for MMD, plays a significant role in TC response by affecting antigen uptake, processing and presentation.^39^ In our study, we observed that in PBMCs, the percentages of CD4^+^Tn, CD4^+^Tcm, Treg and CD8^+^Tcm were elevated in MMD patients compared to HCs. However, CD4^+^Te, CD8^+^Tn, CD8^+^Te, CD8^+^Tem and γδT cells showed significant decreases in MMD patients. An elevation in the number of Treg cells with immunosuppressive function coincides with a reduction in the population of cytotoxic TCs responsible for killing. A prior investigation demonstrated an imbalance of circulating Treg/Th17 cells in patients with MMD.^32^ Weng et al. reported elevated frequencies of Th17 and Treg cells in MMD patients compared to HCs. However, the increased Treg cells observed in MMD patients, particularly those enriched with FrIII Treg cells, exhibited deficient suppressive functions. Our findings align with this prior research.

HLA‐DR expression on TCs is indicative of TC activation. In this study, we observed a significant upregulation of HLA‐DR in TCs, including all T subgroups, indicating a widespread activation of TCs in patients with MMD. Previous study found that HLAs are associated with MMD at the DNA level.^40^, ^41^ Our findings go one step further to illustrate such associations. In the CyTOF cohort, we found a notable enhancement of p‐STAT3 in MMD patient. STAT3 has a crucial role in the development of inflammatory responses and is closely associated with the pathogenesis of autoimmune diseases, such as inflammatory bowel disease and rheumatoid arthritis. Moreover, the strong correlation of p‐STAT3 expression with disease severity suggests that the immune alterations observed in MMD share similarities with those found in autoimmune diseases. Previous studies also demonstrated the activation of STAT3 in neurological diseases, particularly in cerebral ischemic and haemorrhagic stroke.^42^ Therefore, STAT3 may be an important therapeutic target for MMD.

The study emphasized the notable increase in Treg cells in MMD patients, underscoring their crucial role in regulating inflammation and maintaining immune tolerance and homeostasis.^43^ Studies have reported that Treg cells are related to autoimmune diseases, such as multiple sclerosis, systemic lupus erythematosus and rheumatoid arthritis.^23^, ^44^ Recent research on MMD has made significant strides in identifying potential genetic and immunological factors in its pathogenesis.^9^, ^31^, ^45^ Inflammation seems to promote the onset and progression of MMD. Furthermore, impaired Treg cell function has been associated with the occurrence and development of cerebrovascular diseases, including stroke.^46^, ^47^ Treg cell dysfunction can exacerbate inflammatory responses, contributing to the pathogenesis of cerebrovascular diseases and worsening neurological damage.^47^ Furthermore, Treg cells secrete pro‐angiogenic factors that promote uncontrolled angiogenesis and incomplete vascular development in tumours,^48^, ^49^ which may suggest that Treg cells may be associated with angiogenesis in MMD. Previous studies have suggested potential involvement of RNF213 abnormalities in the differentiation of Tregs, potentially compromising immunological self‐tolerance and contributing to the development of MMD.^30^

BCs are closely related to chronic inflammation, extending beyond their conventional role in antibody production. By presenting autoantigens to autoreactive TCs and releasing proinflammatory molecules, BCs actively contribute to the development and advancement of chronic inflammatory diseases. In our study, we observed that MMD patients exhibited an increased percentage of BCs, along with enhanced migration and adhesion capacities of these cells, accompanied by higher expression levels of CXCR3, CX3CR1, CD11b and CD11c. These findings indicate that BCs are closely related to the pathogenesis of MMD. Prior research has also pointed towards the involvement of BCs during central nervous system pathology, including after ischemic and haemorrhagic strokes, further supporting their significance in MMD.^50^ Additionally, a previous study using CIBERSORT reported increased levels of naive BCs in MMD patients, which aligns with our results.^51^ Inflammation is also affected by cytotoxic immune cells, which can be classified into two types: those participating in innate (such as γδT cells, NK cells and NKT cells) and those associated with acquired immunity (CD8^+^T cells). Notably, our study revealed a reduction in the proportions of cytotoxic cells engaged in natural immunity, including γδT and NK cells, in individuals with MMD. This result aligns with a prior bioinformatics analysis that similarly noted diminished NK cell levels in MMD patients.^51^ Despite the diminished numbers of cytotoxic immune cells in individuals with MMD, we observed that NK cells demonstrated heightened migration and adhesion capabilities, coupled with increased expression of CXCR3, CX3CR1, CD11b and CD11c.

RNF213 exhibits substantial upregulation in both DCs and MCs, indicating its pivotal role in the development and functionality of these cellular components.^39^ In RNF213‐knockout mice and mice with knock‐in polymorphisms associated MMD, DCs exhibited compromised ability in antigen uptake, processing and presentation. These deficiencies likely contribute to autoimmune reactions by disrupting the delicate equilibrium between immunity and tolerance.^39^ In this study, we observed a reduction in DCs among MMD patients. Nonetheless, these cells exhibited enhanced migration and adhesion capabilities, accompanied by increased expression of CXCR3, CX3CR1 and CD11b. Notably, TGFβ levels were higher in the DCs of MMD patients. Circulating MCs are thought to contribute to the development of MMD.^52^ In this study, we observed elevated levels of MCs in MMD patients, consistent with a prior bioinformatics study.^11^ Additionally, the migration and adhesion capacities of these cells are enhanced, accompanied by increased expression of CXCR3, CX3CR1, CD11b and CD11c.

Additionally, we found that the EndMT score is higher in patients compared to HCs. EndMT is a process where endothelial cells, which form the inner lining of blood vessels, lose their characteristic features and acquire mesenchymal properties.^53^ EndMT is a physiological occurrence in embryonic development, but it also assumes a pathological role in diverse diseases, such as cardiovascular disease, cancer, fibrosis and inflammation.^53^ EndMT has been extensively studied in cardiovascular disease, where it contributes to the pathogenesis of atherosclerosis, pulmonary hypertension and cardiac fibrosis.^54^ In atherosclerosis, EndMT is implicated in the formation of new blood vessels within the plaque, which can lead to plaque instability and rupture.^55^ In pulmonary hypertension, EndMT contributes to the remodelling of the pulmonary vasculature, resulting in heightened vascular resistance and right heart failure.^56^ In cardiac fibrosis, EndMT is implicated in the build‐up of extracellular matrix proteins in the heart, causing impaired cardiac function.^57^ In our study, TGFβ1 was significantly increased in MMD patients compared to HCs. Targeting molecules involved in EndMT could potentially prevent or reverse the pathological changes associated with this process, offering new hope for MMD patients.

We also note that genetic risk is a common feature of intracranial aneurysms, arteriovenous malformations (AVMs), cavernous malformations and MMD, and familial cases are more likely to occur in the same family. Through pedigree and gene association studies, the association of some possible genetic variants with the pathogenesis of these diseases has been preliminarily confirmed. Several studies have suggested that intracranial aneurysms, AVMs, cavernous malformations and MMD may share certain genes and signalling pathways. For example, some studies have found that mutations in connective tissue‐related genes, such as COL4A1 and COL4A2, are associated with the pathogenesis of intracranial aneurysms.^58^, ^59^, ^60^, ^61^ PI3K‐mTOR signalling is a key pathway downstream of cavernous malformations; similarly, this signalling pathway has been widely reported in AVMs.^62^, ^63^ Interestingly, the results of our GSEA analysis also showed significant enrichment of these pathways during the evolution of MMD peripheral immunity. The increased adoption of genome‐wide association studies and next‐generation sequencing has advanced research, uncovering potential shared genes associated with intracranial aneurysms, AVMs and MMD. These genes are often involved in the integrity of the blood vessel wall, cell proliferation, vascular development and other aspects.^64^, ^65^ These investigations enhance our comprehension of the genetic mechanisms underlying MMD and offer targeted avenues for future strategies in prevention, diagnosis and treatment.

This study has several limitations that need to be acknowledged. First, the study had a limited sample size, and all patients were recruited from a single centre, possibly introducing selection bias and restricting the generalizability of the findings. Second, our study did not include cell or animal experiments to further validate the functions of immune cells. In future investigations, it will be crucial to conduct experiments targeting the identified intriguing clusters to elucidate the underlying mechanisms responsible for the observed alterations in circulating immune cells in MMD.

## 5 CONCLUSION

We presented a comprehensive overview of the circulating immune cell landscape in MMD patients, revealing dysregulated immune functions in PBMCs, notably in TCs. These findings may offer insights into novel treatment strategies for MMD.

## AUTHOR CONTRIBUTIONS

Jizong Zhao, Dong Zhang, Hao Li and Peicong Ge designed the project. Chuming Tao, Peicong Ge, Wenjing Wang, Qiheng He, Chenglong Liu, Zhiyao Zheng, Siqi Mou and Bojian Zhang performed data analysis. Peicong Ge and Chuming Tao wrote the manuscript. Xingju Liu, Qian Zhang, Rong Wang and Yan Zhang revised manuscript. All authors read and approved the final manuscript.

## CONFLICT OF INTEREST STATEMENT

The authors declare that they have no conflicts of interest.

## FUNDING INFORMATION

National Natural Science Foundation of China, Award Numbers: 81891004 and 82301451

## ETHICS APPROVAL AND CONSENT TO PARTICIPATE

The study was reviewed and approved by the Ethics Committee of Beijing Tiantan Hospital, Capital Medical University (KY2022‐051‐02).

## Supporting information

Supporting Information

Supporting Information

Supporting Information

Supporting Information

Supporting Information

Supporting Information

Supporting Information

Supporting Information

Supporting Information

Supporting Information

Supporting Information

Supporting Information

Supporting Information

Supporting Information

Supporting Information

## Data Availability

The raw sequence data reported in this paper have been deposited in the Genome Sequence Archive (Genomics, Proteomics & Bioinformatics 2021) in National Genomics Data Center (Nucleic Acids Res 2022), China National Center for Bioinformation/Beijing Institute of Genomics, Chinese Academy of Sciences (mRNA: HRA004479;) that are publicly accessible at https://ngdc.cncb.ac.cn/gsa‐human. The CyTOF data reported in this paper have been deposited in the OMIX, China National Center for Bioinformation / Beijing Institute of Genomics, Chinese Academy of Sciences (https://ngdc.cncb.ac.cn/omix: accession no. OMIX004669).
