# Supplementary figures and images for: Circulating immune cell landscape and T‐cell abnormalities in patients with moyamoya disease
Source: Clin Transl Med. 2024 Apr 2;14(4):e1647. doi: 10.1002/ctm2.1647 (PMC10988118; doi:10.1002/ctm2.1647)

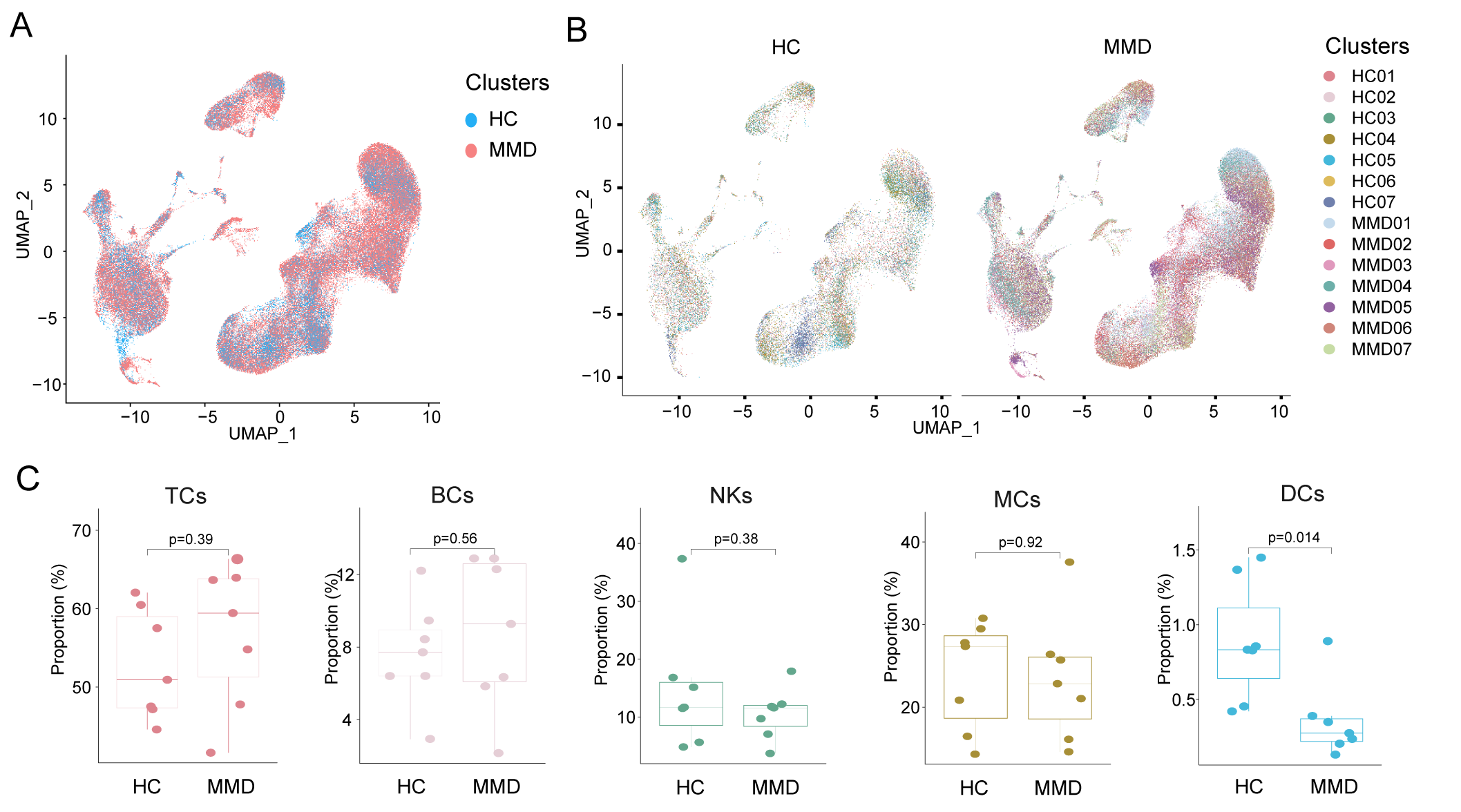

Supplement: Supplementary file 1 — Supporting Information [file CTM2-14-e1647-s002.tif]

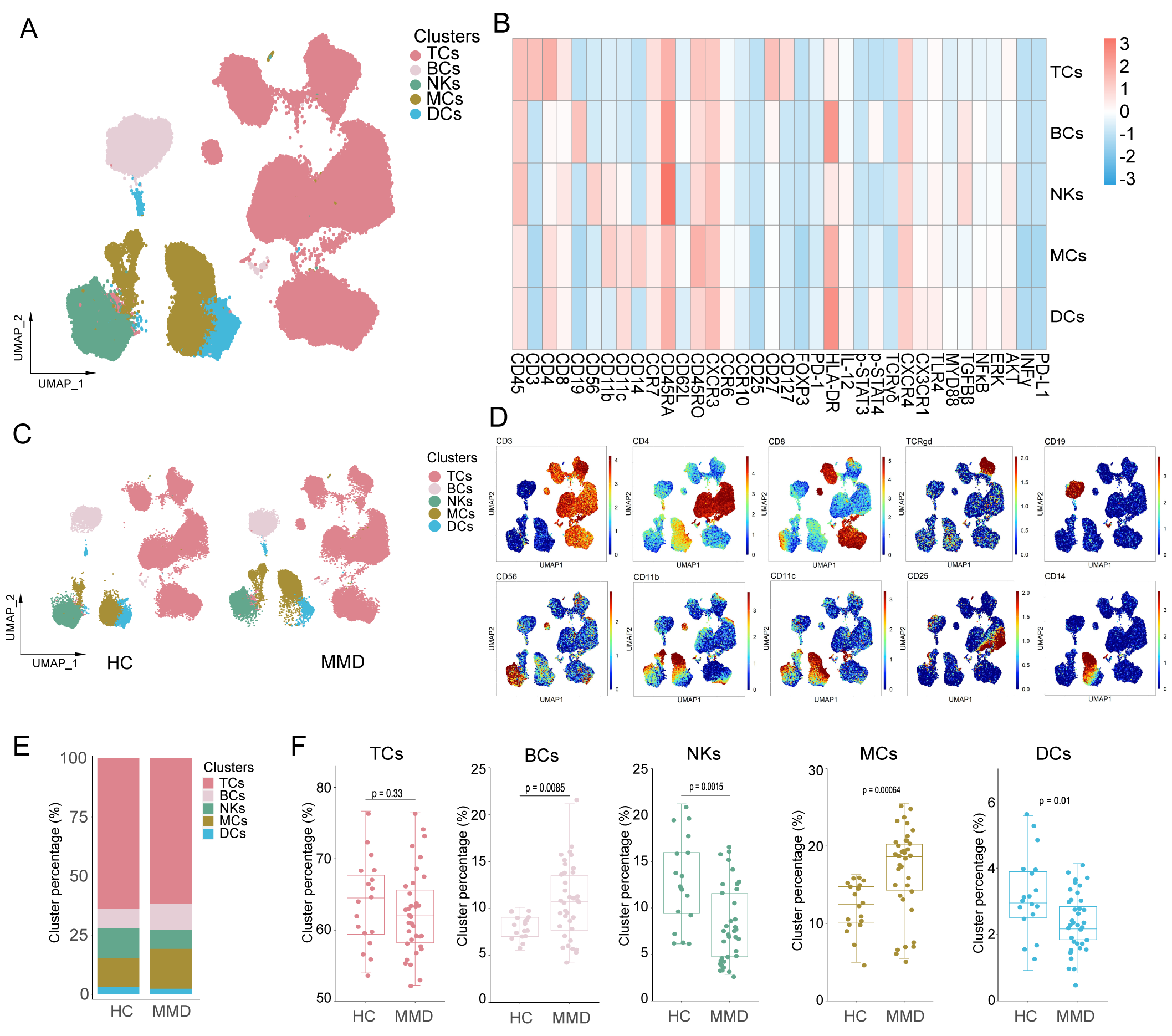

Supplement: Supplementary file 2 — Supporting Information [file CTM2-14-e1647-s014.tif]

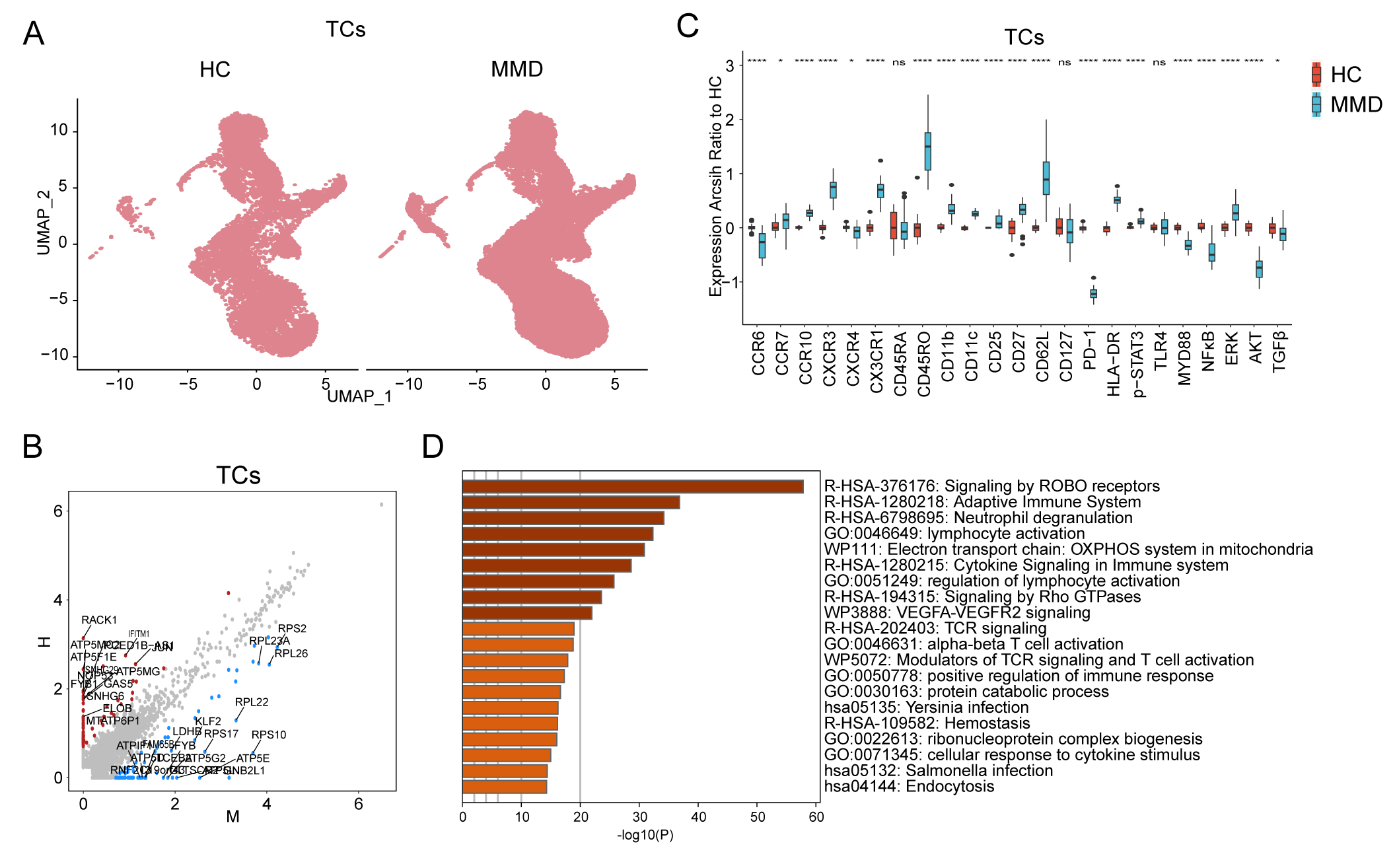

Supplement: Supplementary file 3 — Supporting Information [file CTM2-14-e1647-s015.tif]

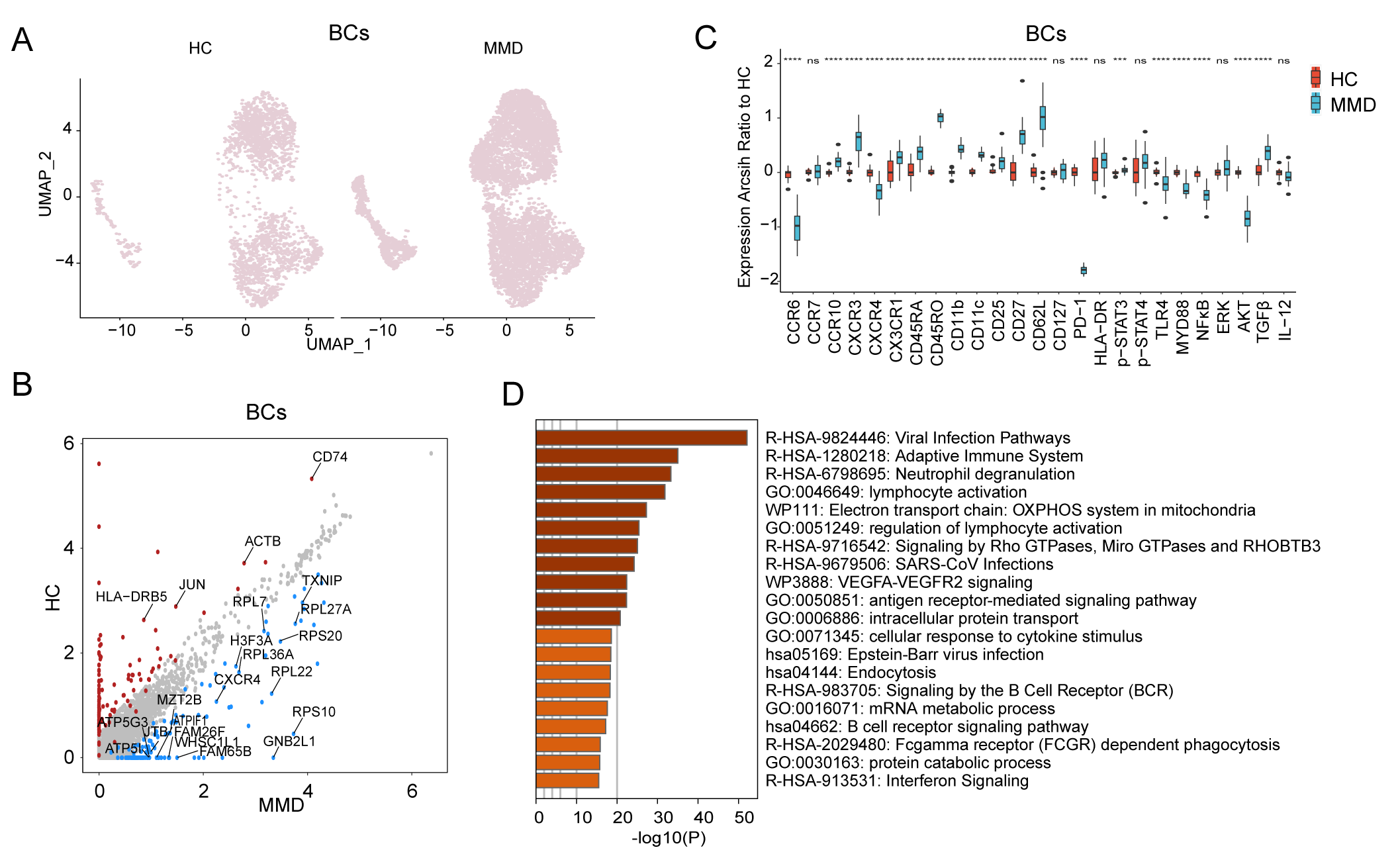

Supplement: Supplementary file 4 — Supporting Information [file CTM2-14-e1647-s010.tif]

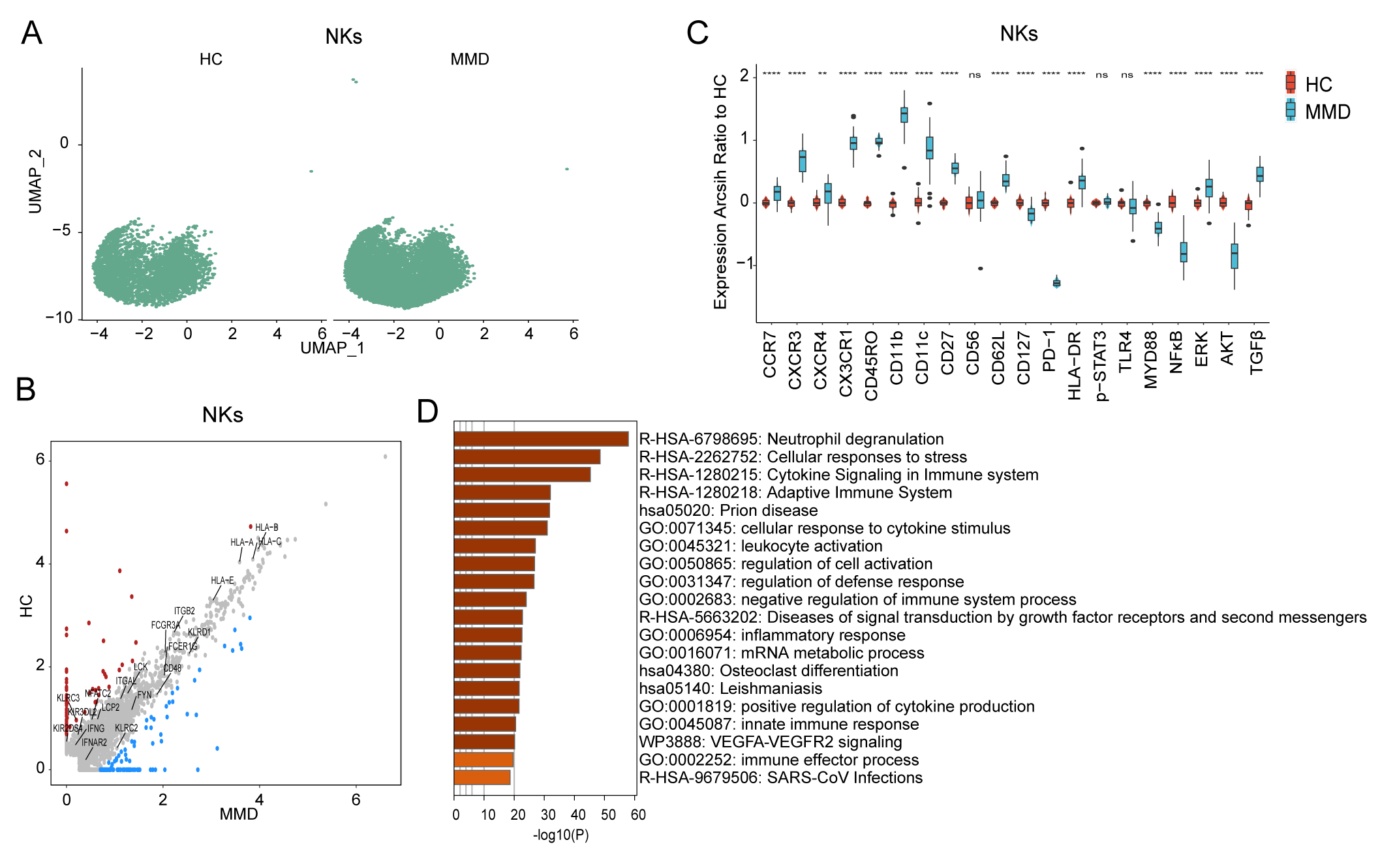

Supplement: Supplementary file 5 — Supporting Information [file CTM2-14-e1647-s011.tif]

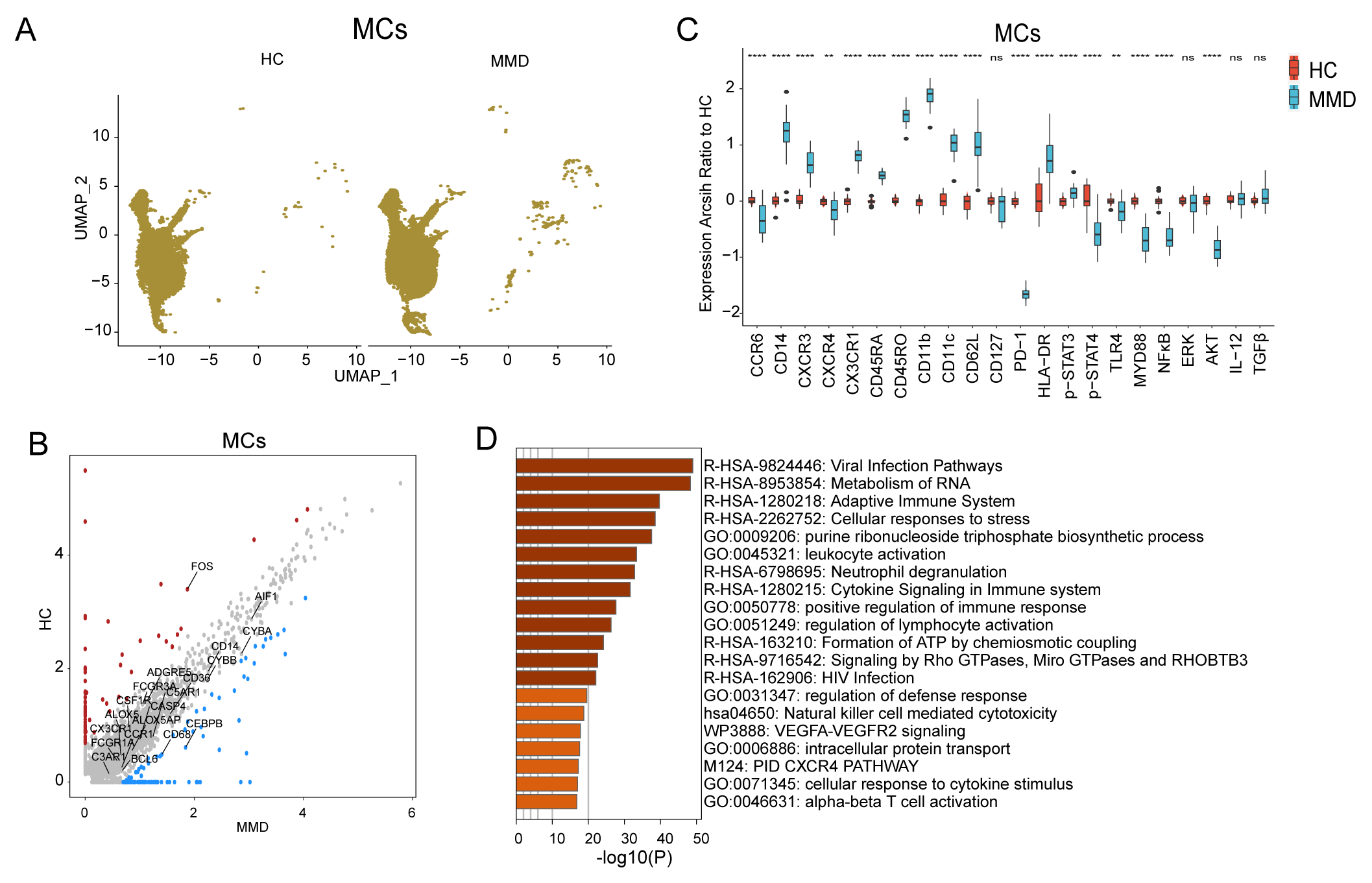

Supplement: Supplementary file 6 — Supporting Information [file CTM2-14-e1647-s004.tif]

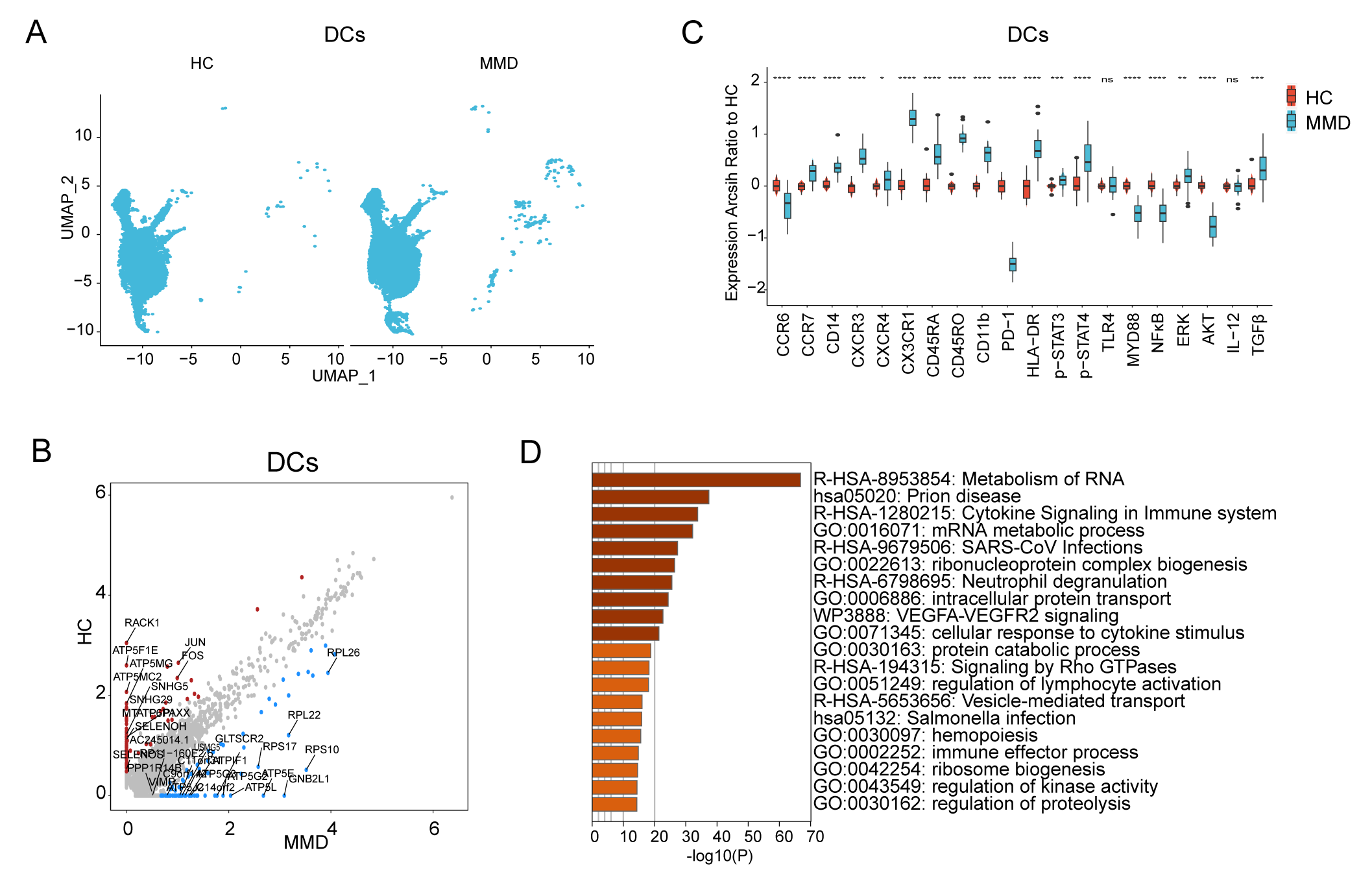

Supplement: Supplementary file 7 — Supporting Information [file CTM2-14-e1647-s008.tif]

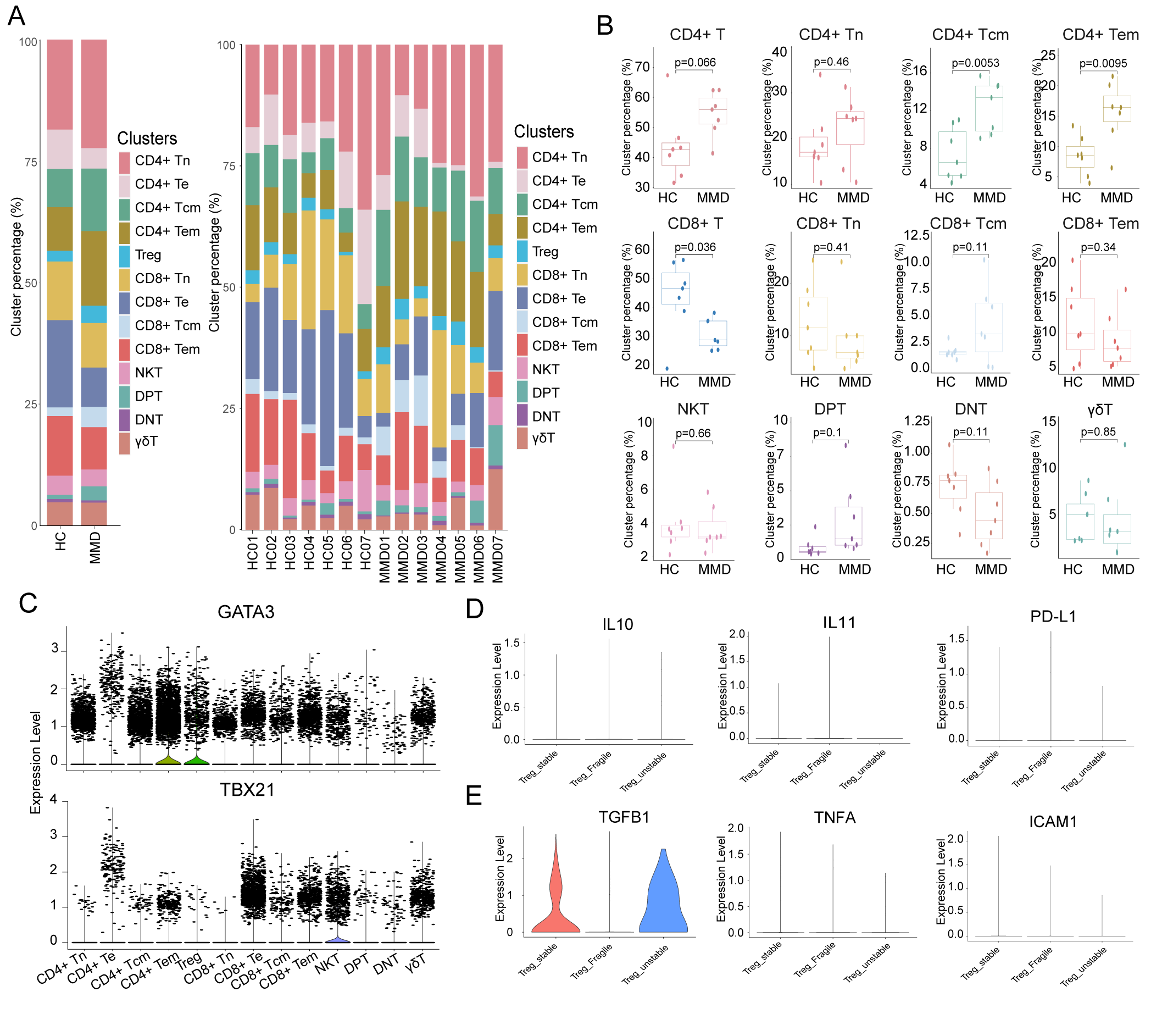

Supplement: Supplementary file 8 — Supporting Information [file CTM2-14-e1647-s006.tif]

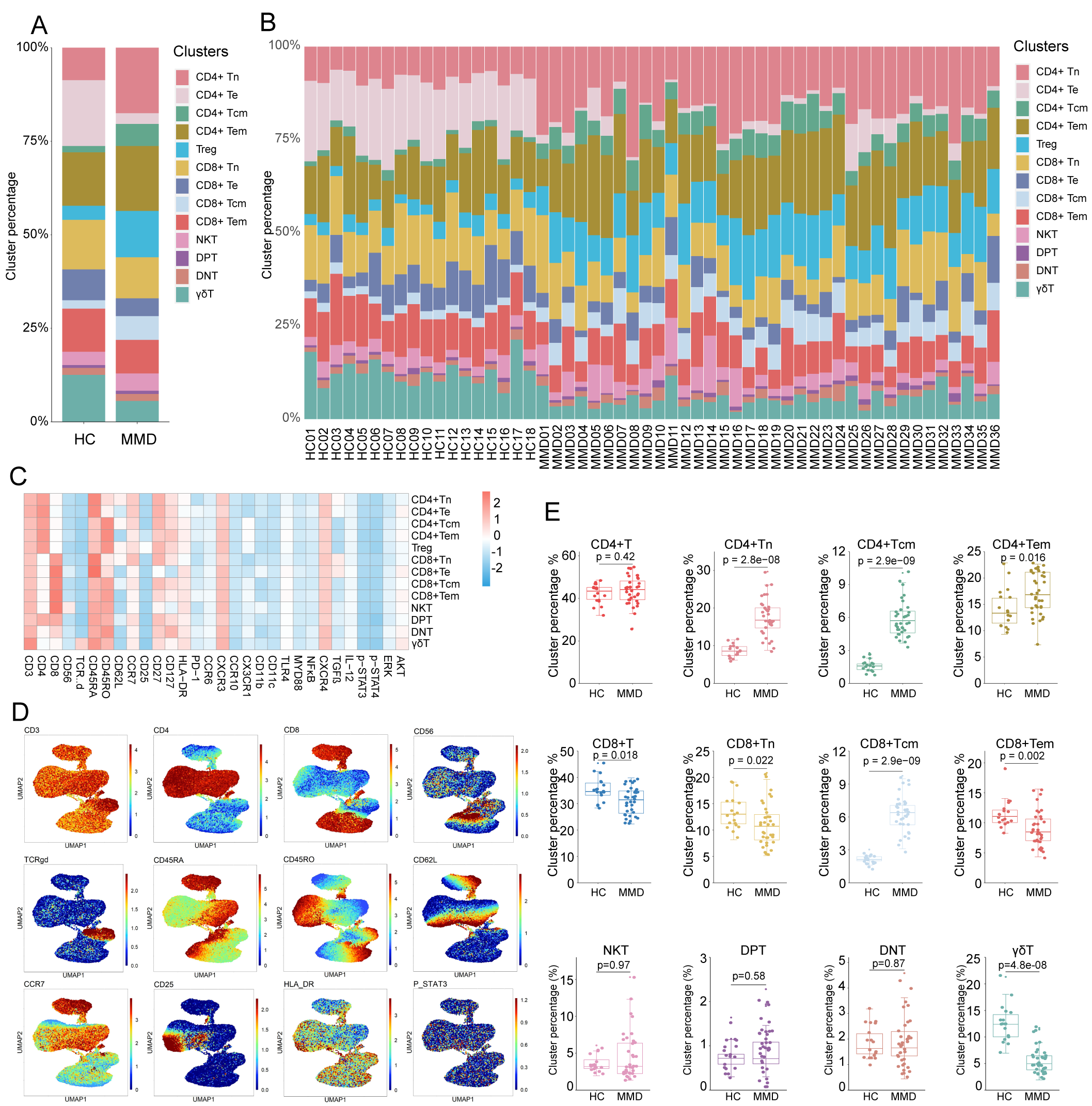

Supplement: Supplementary file 9 — Supporting Information [file CTM2-14-e1647-s003.tif]

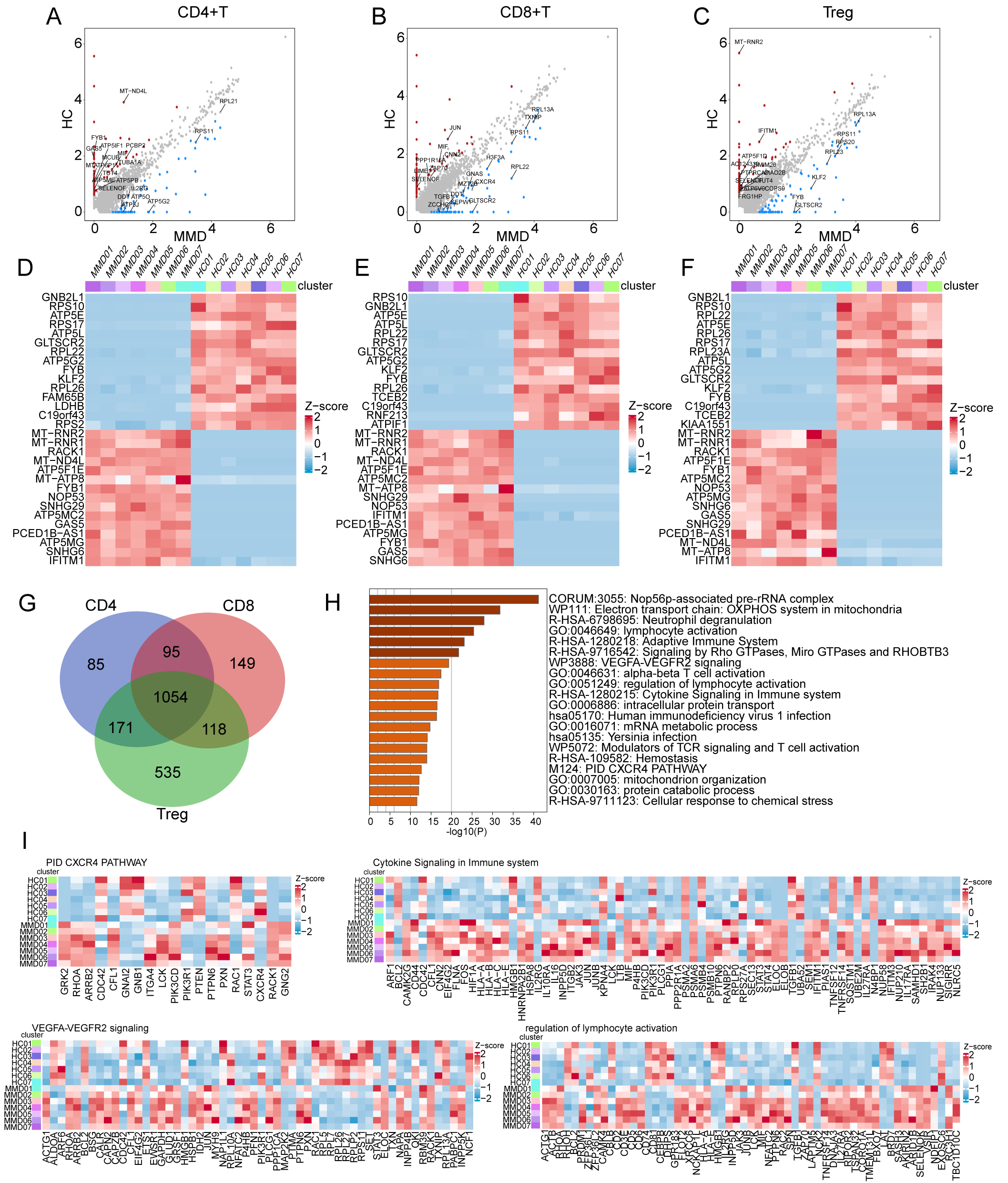

Supplement: Supplementary file 10 — Supporting Information [file CTM2-14-e1647-s005.tif]

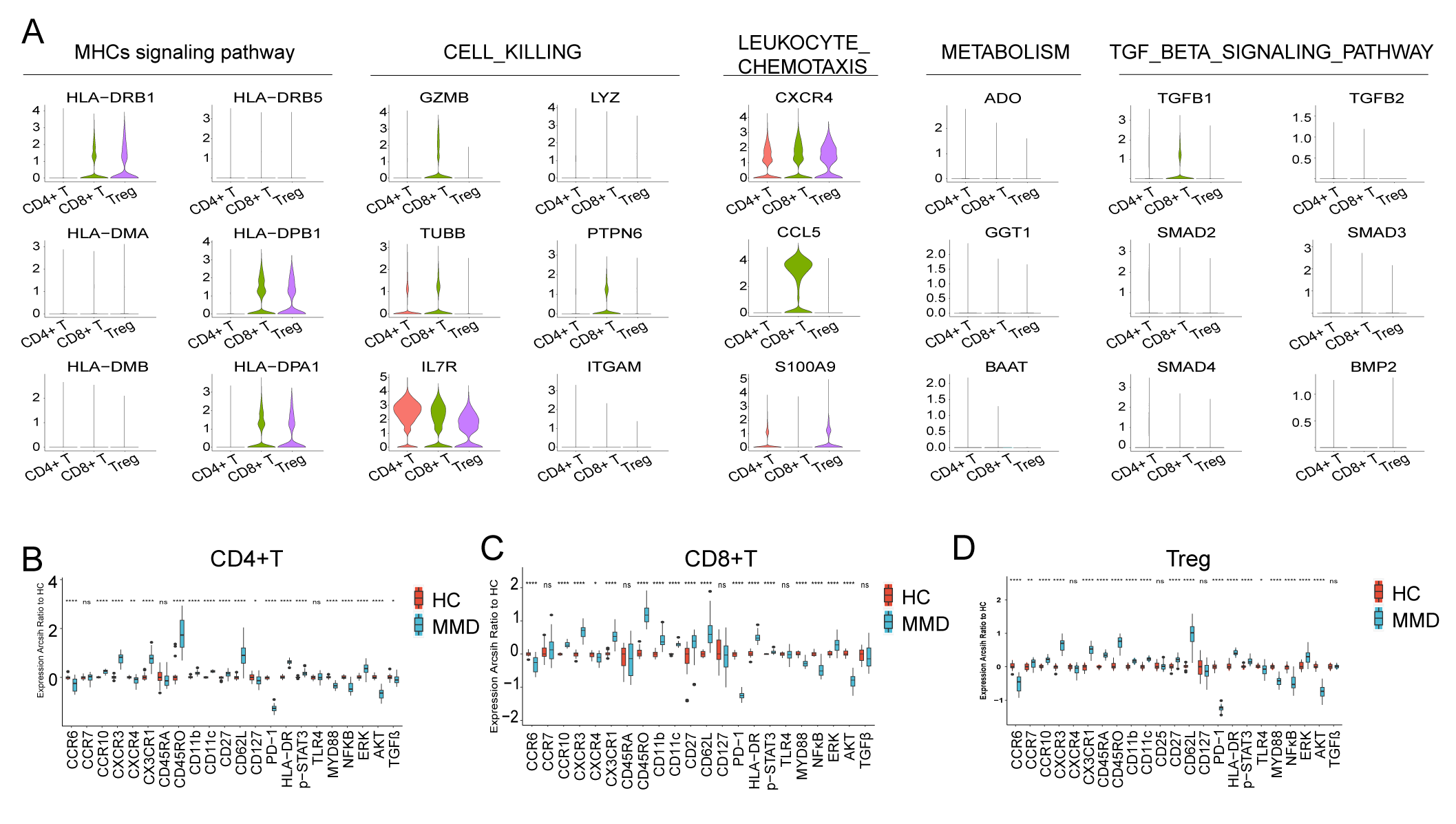

Supplement: Supplementary file 11 — Supporting Information [file CTM2-14-e1647-s013.tif]

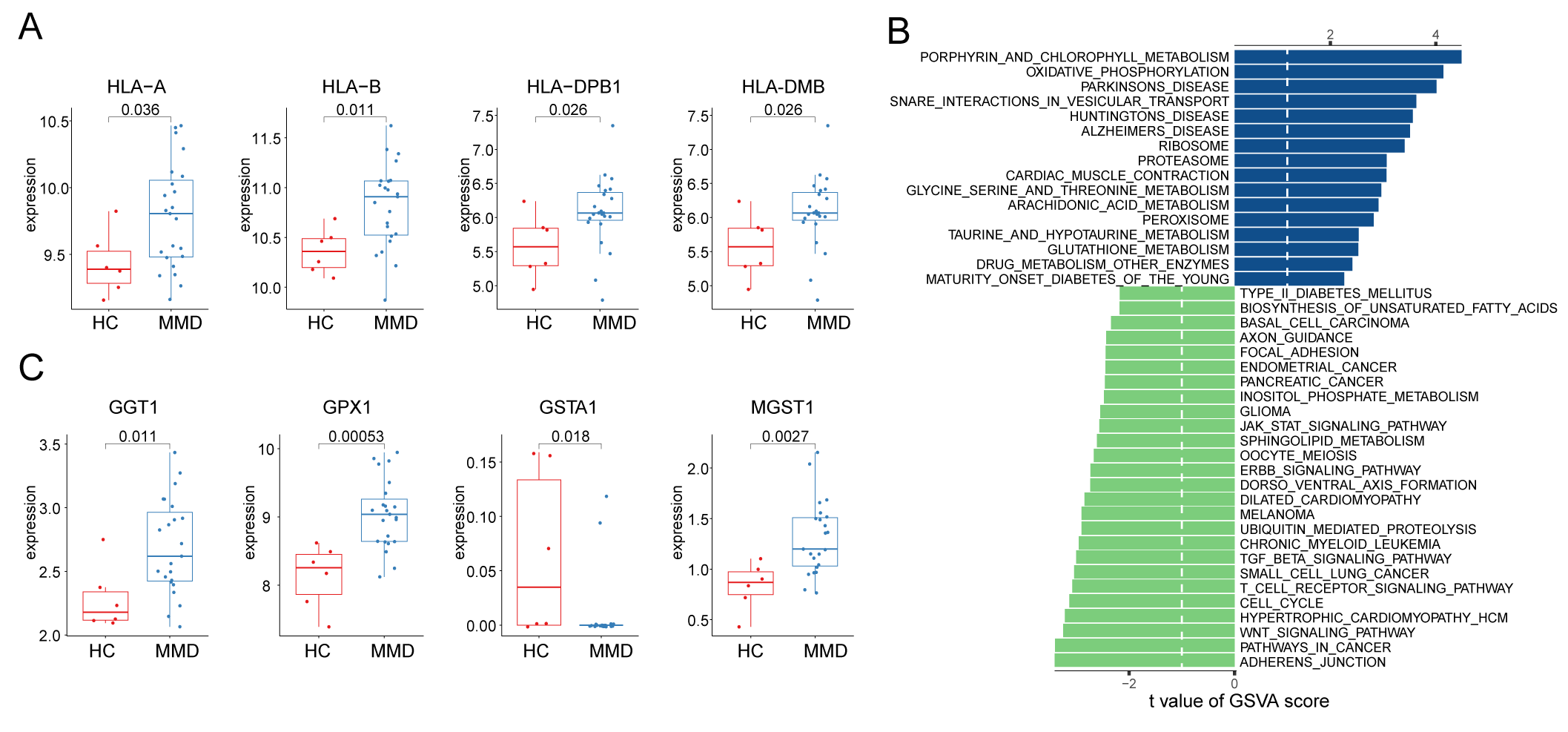

Supplement: Supplementary file 12 — Supporting Information [file CTM2-14-e1647-s009.tif]

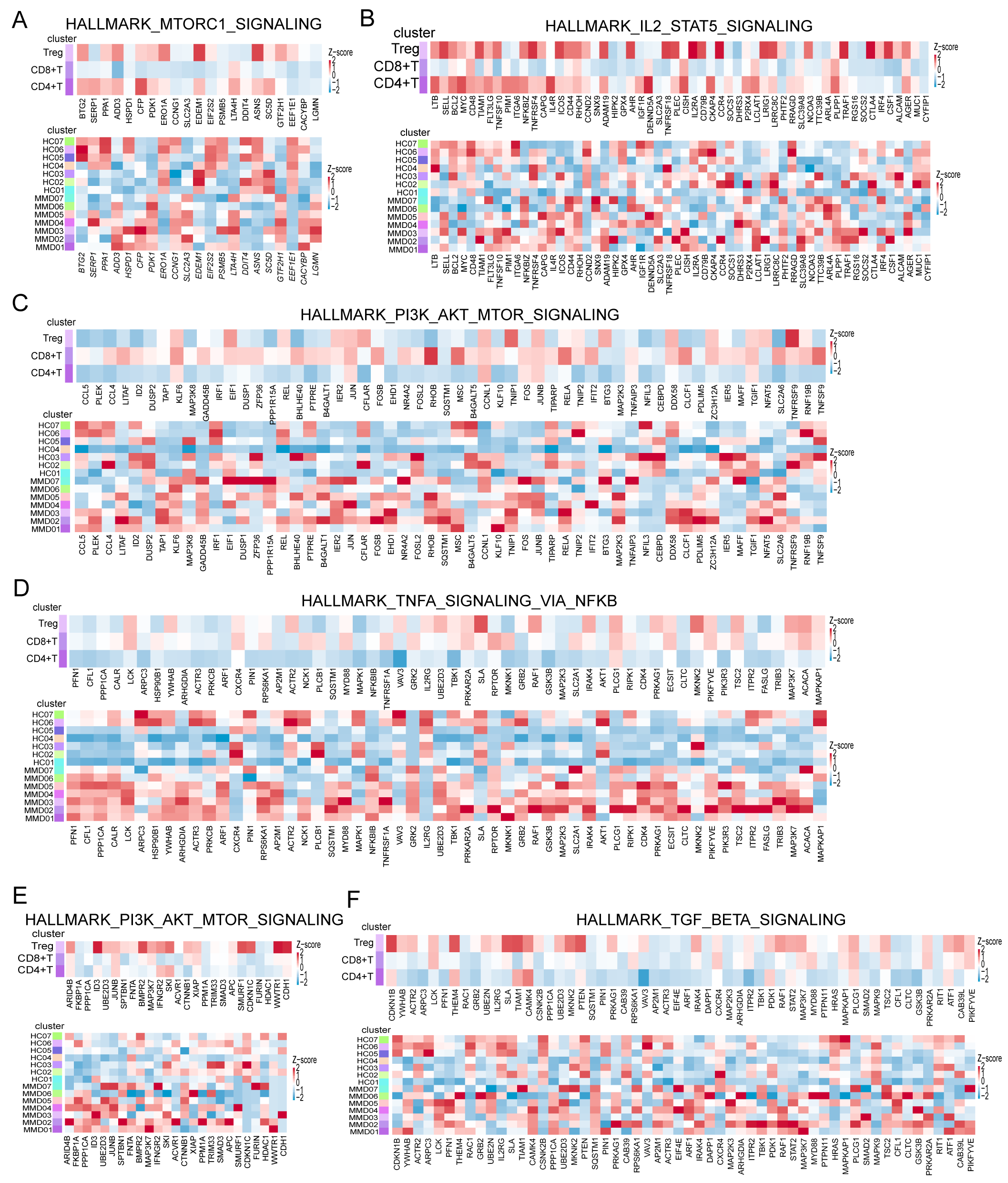

Supplement: Supplementary file 13 — Supporting Information [file CTM2-14-e1647-s012.tif]
