# Supplementary material for: Circulating immune cell landscape and T‐cell abnormalities in patients with moyamoya disease
Source: Clin Transl Med. 2024 Apr 2;14(4):e1647. doi: 10.1002/ctm2.1647 (PMC10988118; doi:10.1002/ctm2.1647)
Supplement: Supplementary file 14 — Supporting Information [file CTM2-14-e1647-s001.docx]

Supplementary Table 1. Clinical Characteristics in Patients With MMD in scRNA-seq cohort.

| Characteristics | MMD Patients  (N=7) | HC  (N=7) |
| --- | --- | --- |
| Demographic |  |  |
| Age, years | 41.9± 5.5 | 40.3± 5.8 |
| Sex, female | 3 (42.9) | 3 (42.9) |
| Current cigarette smoking, n (%) | 1 (14.3) | NA |
| Current alcohol drinking, n (%) | 1 (14.3) | NA |
| Medical history |  |  |
| History of hypertension, n (%) | 2 (28.6) | NA |
| History of hyperlipidemia, n (%) | 2 (28.6) | NA |
| History of diabetes mellitus, n (%) | 0 (0.0) | NA |
| History of thyroid disease, n (%) | 0(0.0) | NA |
| Clinical manifestation |  |  |
| Ischemic type | 4 (57.1) | NA |
| Hemorrhagic type | 3 (42.9) | NA |
| Suzuki Stage |  |  |
| 1-2 | 1 (14.3) | NA |
| 3-4 | 6 (85.7) | NA |

Supplementary Table 2. Clinical Characteristics in Patients With MMD and HCs in CyTOF cohort.

| Characteristics | HCs  (N=18) | MMD Patients  (N=36) | *t/Z* | *P*-value |
| --- | --- | --- | --- | --- |
| Demographic |  |  |  |  |
| Age, years | 35.2±7.9 | 38.9±7.2 | -0.323 | 0.747 |
| Sex, female | 9 (52.9) | 19 (52.8) | 0.037 | 0.847 |
| Current cigarette smoking, n (%) | 0 (0.0) | 10 (27.8) | - | 0.021 |
| Current alcohol drinking, n (%) | 0 (0.0) | 5 (13.9) | - | 0.157 |
| Medical history |  |  |  |  |
| History of hypertension, n (%) | 0 (0.0) | 12 (33.3) | - | 0.005 |
| History of hyperlipidemia, n (%) | 0 (0.0) | 5 (13.9) | - | 0.157 |
| History of diabetes mellitus, n (%) | 0 (0.0) | 1 (2.8) | 0.000 | 1.000 |
| History of thyroid disease, n (%) | 0 (0.0) | 0(0.0) | - | 1.000 |
| Clinical manifestation |  |  |  |  |
| Ischemic type | - | 20 (55.6) |  |  |
| Infarction | - | 12 (33.3) |  |  |
| TIA | - | 8 (22.2) |  |  |
| Hemorrhagic type | - | 16 (44.4) |  |  |
| IVH | **-** | 11 (30.6) |  |  |
| ICH | **-** | 3 (8.3) |  |  |
| ICH+IVH | - | 1 (2.8) |  |  |
| SAH | - | 1 (2.8) |  |  |
| Suzuki Stage |  |  |  |  |
| 1-2 | - | 6 (16.7) |  |  |
| 3-4 | - | 24 (66.7) |  |  |
| 5-6 | - | 6 (16.7) |  |  |

ICH, intracranial haemorrhage; IVH, intraventricular haemorrhage; SAH, subarachnoid haemorrhage; TIA, transient ischaemic attack.

Supplementary Table 3. Clinical Characteristics in Patients With MMD and HCs in RNA-seq cohort.

| Characteristics | HCs  (N=6) | MMD Patients  (N=23) | *t/Z* | *P*-value |
| --- | --- | --- | --- | --- |
| Demographic |  |  |  |  |
| Age, years | 30.0±8.3 | 31.1±8.5 | 0.829 | 0.854 |
| Sex, female | 4 (66.7) | 15 (65.2) | 0.000 | 1.000 |
| Current cigarette smoking, n (%) | 0 (0.0) | 1 (4.3) | - | 1.000 |
| Current alcohol drinking, n (%) | 0 (0.0) | 3 (13.0) | - | 1.000 |
| Medical history |  |  |  |  |
| History of hypertension, n (%) | 0 (0.0) | 7 (30.4) | - | 0.289 |
| History of hyperlipidemia, n (%) | 0 (0.0) | 3 (13.0) | - | 1.000 |
| History of diabetes mellitus, n (%) | 0 (0.0) | 1 (4.0) | - | 1.000 |
| History of thyroid disease, n (%) | 0 (0.0) | 1 (4.0) | - | 1.000 |
| Clinical manifestation |  |  |  |  |
| Ischemic type | - | 12 (52.2) |  |  |
| Hemorrhagic type | - | 11 (47.8) |  |  |
| Suzuki Stage |  |  |  |  |
| 1-2 | - | 8 (34.8) |  |  |
| 3-4 | - | 11 (47.8) |  |  |
| 5-6 | - | 4 (17.4) |  |  |

ICH, intracranial haemorrhage; IVH, intraventricular haemorrhage; SAH, subarachnoid haemorrhage; TIA, transient ischaemic attack.

Supplementary Table 4. Clinical Characteristics in Patients With MMD and HCs.

| Characteristics | HCs  (N=34) | MMD Patients  (N=120) | *t/Z* | *P*-value |
| --- | --- | --- | --- | --- |
| Demographic |  |  |  |  |
| Age, years | 35.2±7.9 | 36.4±8.4 | -0.758 | 0.449 |
| Sex, female | 18 (52.9) | 76 (63.3) | 1.203 | 0.273 |
| Current cigarette smoking, n (%) | 0 (0.0) | 17 (14.2) | - | 0.025 |
| Current alcohol drinking, n (%) | 0 (0.0) | 13 (10.8) | - | 0.073 |
| Medical history |  |  |  |  |
| History of hypertension, n (%) | 0 (0.0) | 27 (22.5) | - | 0.001 |
| History of hyperlipidemia, n (%) | 0 (0.0) | 12 (10.0) | **-** | 0.070 |
| History of diabetes mellitus, n (%) | 0 (0.0) | 10 (8.3) | **-** | 0.119 |
| History of thyroid disease, n (%) | 0 (0.0) | 3(1.9) |  | 1.000 |
| Clinical manifestation |  |  |  |  |
| Ischemic type | - | 80 (66.7) |  |  |
| Infarction | - | 36 (30.0) |  |  |
| TIA | - | 44 (36.7) |  |  |
| Hemorrhagic type | - | 40 (33.3) |  |  |
| IVH | **-** | 23 (19.2) |  |  |
| ICH | **-** | 9 (7.5) |  |  |
| ICH+IVH | - | 6 (5.0) |  |  |
| SAH | - | 2 (1.7) |  |  |
| Suzuki Stage |  |  |  |  |
| 1-2 | - | 16 (13.3) |  |  |
| 3-4 | - | 81 (67.5) |  |  |
| 5-6 | - | 6 (5.0) |  |  |

ICH, intracranial haemorrhage; IVH, intraventricular haemorrhage; SAH, subarachnoid haemorrhage; TIA, transient ischaemic attack.
