# Supplementary material for: Circulating immune cell landscape and T‐cell abnormalities in patients with moyamoya disease
Source: Clin Transl Med. 2024 Apr 2;14(4):e1647. doi: 10.1002/ctm2.1647 (PMC10988118; doi:10.1002/ctm2.1647)
Supplement: Supplementary file 15 — Supporting Information [file CTM2-14-e1647-s007.docx]

**FIGURE S1** Single-cell transcriptional profiling.

(A) The UMAP projection of PBMCs from the HCs and MMDs, groups colored by group information. (B) The UMAP projection of PBMCs from from the 7 MMDs and 7 HCs samples colored by group information. (C) Percentage of 5 subsets cells in PBMCs from HCs and MMDs groups.

**FIGURE S2** Single-cell CyTOF analysis of peripheral immune cells.

(A) The UMAP projection visualization of PBMC 5 cell subsets in CyTOF cohort. (B) The heatmap shows the expression of the each markers in CyTOF cohort to identify the cell subset. (C) The UMAP projection of PBMCs in HCs(C) and MMD (D) groups derived from CyTOF cohort. (D) Cell markers are used to identify and verify the distribution of each cell subset in CyTOF cohort. (E) The proportion of each cell subset as well as its distribution in CyTOF cohort. (F) Percentage of 5 subsets cells in PBMCs from HCs and MMDs groups in CyTOF cohort.

**FIGURE S3** Changes in TCs function in patients with MMD.

(A) The UMAP projection visualization of TCs subset in scRNA-seq cohort. (B) The volcano plot show the DEGs in the TCs between the MMD and HC groups. (C) In the CyTOF cohort, the expression of some cytokines between the MMD and HC groups in the TCs. (D) DEGs of TCs were enriched to show changes in cell function and pathways.

**FIGURE S4** Changes in BCs function in patients with MMD.

(A) The UMAP projection visualization of BCs subset in scRNA-seq cohort. (B) The volcano plot show the DEGs in the BCs between the MMD and HC groups. (C) In the CyTOF cohort, the expression of some cytokines between the MMD and HC groups in the BCs. (D) DEGs of BCs were enriched to show changes in cell function and pathways.

**FIGURE S5** Changes in NKs function in patients with MMD.

(A) The UMAP projection visualization of NKs subset in scRNA-seq cohort. (B) The DEGs in the NKs between the MMD and HC groups. (C) In the CyTOF cohort, the expression of some cytokines between the MMD and HC groups in the NKs. (D) DEGs of NKs were enriched to show changes in cell function and pathways.

**FIGURE S6** Changes in MCs function in patients with MMD.

(A) The UMAP projection visualization of MCs subset in scRNA-seq cohort. (B) The DEGs in the MCs between the MMD and HC groups. (C) In the CyTOF cohort, the expression of some cytokines between the MMD and HC groups in the MCs. (D) DEGs of MCs were enriched to show changes in cell function and pathways.

**FIGURE S7** Changes in DCs function in patients with MMD.

(A) The UMAP projection visualization of DCs subset in scRNA-seq cohort. (B) The DEGs in the DCs between the MMD and HC groups. (C) In the CyTOF cohort, the expression of some cytokines between the MMD and HC groups in the DCs. (D) DEGs of DCs were enriched to show changes in cell function and pathways.

**FIGURE S8** Detailed annotation of T cell subsets in single-cell cohort.

(A) The proportion of each T cell subset as well as its distribution. (B) Percentage of TCs subsets in PBMCs from HCs and MMDs groups in scRNA-seq cohort. (C) GATA3 and TBX21 expressions in TCs subsets. (D) The expression of inhibitory cytokines in Treg cells subsets. (E) The expression of inflammatory factors in Treg cells subsets.

**FIGURE S9** Re-clustering of T cell subsets derived from CyTOF cohort.

(A, B) The proportion of each T cell subset as well as its distribution. (C) The heatmap shows the expression of each marker in CyTOF cohort to identify the T cell subset. (D) Cell markers are used to identify and verify the distribution of each T cell subset in CyTOF cohort. (E) Percentage of TCs subsets in PBMCs from HCs and MMDs groups in CyTOF cohort.

**FIGURE S10** Changes of T cell subsets in transcriptional profiles from scRNA-seq.

(A) The DEGs between MMD and HC groups in CD4^+^T cells. (B) The DEGs between MMD and HC groups in CD8^+^T cells. (C) The DEGs between MMD and HC groups in Treg cells. (D) Heatmap show the top 30 of the DEGs between MMD and HC groups in CD4^+^T cells. (E) Heatmap show the top 30 of the DEGs between MMD and HC groups in CD8^+^T cells. (F) Heatmap show the top 30 of the DEGs between MMD and HC groups in Treg cells. (G) The Venn diagram shows that 1054 common DEGs were obtained from a subpopulation of 3 T cells. (H) The 1054 DEGs in G were enriched to show changes in cell function and pathways. (I) Heatmap of genes in PID CXCR4 pathway, VEGFA-VEGFR2 signaling, Cytokine Signaling in immune system, and regulation of lymphocyte activation selected from enrichment analyses in H.

**FIGURE S11** The expression of inflammatory factors in CD4^+^T, CD8^+^T, and Treg cells subsets.

(A) Expression of some genes involved in metabolism, cell killing, MHCS signaling pathway, leukocyte chemotaxis and tgf_beta_signaling_pathway, stratified by T cell subpopulation. (B) In the CyTOF cohort, the expression of some cytokines between the MMD and HC groups in the CD4^+^T cell subset. (C) In the CyTOF cohort, the expression of some cytokines between the MMD and HC groups in the CD8^+^T cell subset. (D) In the CyTOF cohort, the expression of some cytokines between the MMD and HC groups in the Treg cell subset.

**FIGURE S12** Changes of RNA-seq cohort in transcriptional profiles.

(A) The expression of some HLA family genes in the RNA-seq dataset. (B) The expression of some metabolism related genes in the RNA-seq dataset. (C) In RNA-seq cohort, GSVA enrichment analysis was performed between MMD and HC groups.

**FIGURE S13** Gene expression of related pathways in GSEA analysis results.

(A, B) Heatmap of genes in MTORC1_SIGNALING (A) and IL2_STAT5_SIGNALING (B) pathway selected from GSEA analysis in CD4^+^T subset. (C, D) Heatmap of genes in PI3K_AKT_MTOR_SIGNALING (C) and TNFA_SIGNALING_VIA_NFKB pathway (D) selected from GSEA analysis in CD8^+^T subset. (E, F) Heatmap of genes in PI3K_AKT_MTOR_SIGNALING (E) and TGF_BETA_SIGNALING (F) pathway selected from GSEA analysis in Treg subset.
